# Exploring chaos and ergodic behavior of an inductorless circuit driven by stochastic parameters

**DOI:** 10.1007/s11071-024-10050-x

**Published:** 2024-08-05

**Authors:** Soumyajit Seth, Abhijit Bera, Vikram Pakrashi

**Affiliations:** 1 UCD Centre for Mechanics, Dynamical Systems and Risk Laboratory, School of Mechanical and Materials Engineering, University College Dublin, Dublin, Ireland; 2 School of Technology Management and Engineering, NMIMS Hyderabad, Jadcherla Campus, Raipalle, 509301 India; 3 Department of Physical Sciences, Indian Institute of Science Education and Research Kolkata, Mohanpur Campus, Kalyani, IN 741246 India; 4 UCD Centre for Mechanics, Dynamical Systems and Risk Laboratory, School of Mechanical and Materials Engineering, University College Dublin, Dublin, Ireland

**Keywords:** Ergodicity, Chaos, Lyapunov Exponent, Switching Dynamical Systems, Piecewise-smooth map, Switching Electronic Circuit.

## Abstract

There exist extensive studies on periodic and random perturbations of various smooth maps investigating their dynamics. Unlike smooth maps, non-smooth maps are yet to be studied extensively under a stochastic regime. This paper presents a stochastic piecewise-smooth map derived from a simple inductorless switching circuit. The stochasticity is introduced in parameter values. The distribution of the parameter values is bounded and randomly selected from uniform and triangular distributions and ranges between high and low bifurcation parameter values of the deterministic map. Due to this inherent stochasticity in parameter values, the time evolution of the state variable cannot be predicted at a specific time instant. We observe that the state variable exhibits completely ergodic behavior when the minimum value of the parameter is the same as the minimum bifurcation parameter of the deterministic system. However, the ensemble average of the state variable converges to a fixed value. The system demonstrates nonchaotic behavior for a particular range of parameter values but the deterministic map in that bifurcation range shows interplay between chaos and periodic orbits. The values of Lyapunov exponents decrease monotonically with increased asymmetry of the distribution from which the bifurcation parameter values are chosen. We determine the probability density function of the stochastic map and verify its invariance under initial conditions. The most noteworthy result is the disappearance of chaotic behavior when the lower range of the distribution is varied while maintaining a fixed upper threshold for a particular distribution, even though the deterministic map exhibits an array of periodic and chaotic behaviors within the range. As the period-incrementing cascade with chaotic inclusion only occurs in nonsmooth maps, this paper numerically shows the stochasticity of a piecewise-smooth map obtained from a practical system for the first time where randomness is introduced in the parameter space.

## Introduction

In the last four decades, a large class of physical and engineering systems, including power electronics circuits, impacting systems, stick–slip oscillations, walking and hopping mechanisms in robotics, etc., have been portrayed as switching dynamical systems. Under stroboscopic sampling, these non-autonomous switching dynamical systems may give rise to piecewise-smooth maps [1–4]. Such systems exhibit a special class of dynamical phenomena called *‘ Border Collision Bifurcations’* [5–7], where under the variation of one of the parameters, the location of the fixed point changes. At a critical parameter value, it collides with the border. Such an event abruptly changes the stability status of the map, resulting in a rich array of bifurcation phenomena, which do not occur in smooth dynamical systems.

A period-adding sequence is a sequence of border collision bifurcations occurring in one-dimensional switching dynamical systems. When a parameter is varied, the system goes through a series of bifurcations. These types of bifurcations can be classified under four different types: pure-period increment, period-increment with the coexistence of attractors, period-increment with period-adding inclusions, and period-increment with chaotic inclusions [8]. We have considered the fourth one for this study, which has been shown theoretically [9] and experimentally [10] in a simple one-dimensional, non-autonomous, inductorless switching circuit. Under the smooth variation of the input DC voltage, the system goes through a series of bifurcations, resulting in a sequence of periodic windows between chaotic attractors. The periodicity in each window is one greater than that of the previous one [8, 11]. This circuit is significant in practical applications, especially in chaotic communications, because it has no inductor component and can generate robust chaos [12] under specific parameter settings.

Every physical system has inherently different forms of noise, often manifesting as uncontrollable fluctuations within the system. Due to this, the dynamics of the system may deviate from what we expect theoretically without noise. For a circuit, such noise typically arises from factors like thermal effects, parameter variations due to temperature changes or electromagnetic effects. It has been shown that stochasticity, representing this intrinsic randomness, can be introduced into deterministic systems using different methodologies for smooth systems whose functional forms are differentiable everywhere in the state space. Previous works related to stochastic smooth maps obtained from physical systems [13–15] have explored whether the state variables tend to converge to zero over long-term iterations, thereby exhibiting a delta function of state variables at zero during significant time intervals. It has been confirmed that, under certain conditions, this delta function at zero-valued state variables indeed exists.

One approach to introducing stochasticity in the smooth map involves incorporating external noise at each time-point of the Logistic map, as discussed by Erguler et al. [16]. In [17], stochasticity was introduced to the Logistic map by randomly selecting bifurcation parameters from a distribution ranging between lower and upper bifurcation parameter values of the deterministic map. This introduced uncertainties in the state variable of the system due to fluctuations across parameter spaces, initial conditions, etc.

Furthermore, research has explored the impact of random noise in coupled systems [18], revealing phenomena such as noise-induced synchronization [19, 20], interaction between noise and coupling [21, 22], noise-induced transitions [23], and pattern synchronization [24, 25]. These analytical and numerical investigations show deviations in system dynamics from what would be expected purely from deterministic behavior, highlighting the intricate interplay between noise and system behavior.

The abovementioned works are all related to the effect of stochasticity in the maps calculated from smooth dynamical systems. Several works have also focused on the dynamics of piecewise-smooth maps in the presence of stochasticity. A numerical analysis by Simpson and Kuske [26] investigated the effects of small-amplitude, additive, white Gaussian noise on the stable sliding motion for a piecewise-smooth map. In another study by Simpson and Kuske [27], stochastic dynamics near a periodic orbit were examined when a small noise was embedded in a general piecewise-smooth vector field.

Simpson et al. [28] conducted numerical investigations on a specific type of two-dimensional piecewise-smooth map known as the Nordmark map, which gives the square root singularity at the bifurcation point [29]. They introduced additive Gaussian noise with a fixed amplitude and observed irregular behaviors resulting from the noise, causing transitions between different dynamical behaviors as the bifurcation parameter varied. Rounak and Gupta [30] showed numerically the effect of randomness in the forcing function on a harmonically excited bilinear impact oscillator with a soft barrier, which led to qualitatively disparate behaviors at different parameter values, including the presence of multiple coexisting attractors.

It has been numerically shown that the periodic or stochastic variations of the normal form of piecewise-smooth maps can lead to significant changes in their dynamics due to the coexistence of state and time-dependent switchings [31–33] or the stochastic variation of the borders of the functional form of the map obtained from the state equations of the system [34, 35]. The functional form of the map of the switching system changes either periodically or stochastically due to the presence of both kinds of switching in a system. Under the periodic variation of the functional form of the one-dimension map in any one compartment of the phase space, the bifurcation from a stable attractor to another stable attractor of a period greater than the previous one due to border collision bifurcation has been established. When the functional form changes stochastically, the dynamical behavior of any two orbits may differ even if they start from the same initial point inside the non-deterministic basin of attraction. On the other hand, the piecewise-smooth map with a border, varying stochastically inside a small region of the phase space, gives rise to a non-deterministic basin of attraction. Similar to the case of stochastically varying functional forms, the dynamical behavior of any two orbits, starting from the same initial point inside this non-deterministic basin of attraction, may differ.

Existing literature indicates that stochasticity has been widely introduced in smooth maps. There are a few works where stochasticity is incorporated either through the functional forms of piecewise-smooth maps or by introducing randomness at the borders of the map. However, there is a strong need for further studies in piecewise-smooth maps, both in terms of understanding the interaction of stochasticity with the parameters as well as for the dynamical phenomena they exhibit. In particular, such investigation is important for systems with practical applications, and there is a strong gap in the literature around this. In this study, we tried to address this gap and adopted a different approach by introducing stochasticity in the parameter space of the non-smooth map derived from an inductorless chaos generator circuit [9, 36], which has direct applications in communications. The stochasticity in this work is chosen from a probability distribution within a specific parameter range from the work shown in [37]. The probability density function of the state variable is plotted to determine whether the state variable converges to the Maxwell-Boltzmann distribution in the long-time limit for a stochastic map, as discussed in [38] and [39]. By doing so, we aim to explore whether the system exhibits any new dynamical behavior other than the dynamics obtained from the deterministic map when varying the distribution.

Additionally, this approach allows for investigating and uncovering any characteristics in the stochastic map, such as the interplay of periodic and chaotic orbits, that may reveal the presence of deterministic non-smooth map in the parameter regime. Unlike previous approaches, where the input voltage parameter was considered to have a fixed value, this paper considers it a random variable independently chosen from a distribution. It can capture realistic variations in the chosen system and the consequences of such realistic variations in their dynamical responses.

Our paper is structured into three main parts. The first part, detailed in Sect.  2, covers a brief description of the physical system and the functional form of the map. Section 3 outlines the methodology for introducing stochastic elements into the system, while Sect. 4 delves into the concept of ergodicity and its incorporation into the system. We demonstrate the ergodicity of the system by varying parameter values using methods such as the TM and NVN methods, respectively. Section 5 shows the invariant probability density function of the state variable. Furthermore, Sect. 6 provides a comprehensive analysis of the effects of the two methods on the system under both chaotic and non-chaotic regimes across the parameter space. In this section, we also present the analytical results of the TM method obtained from Sect. 5. The paper ends with conclusions in Sect. 7.

## System under investigation

**Fig. 1:**
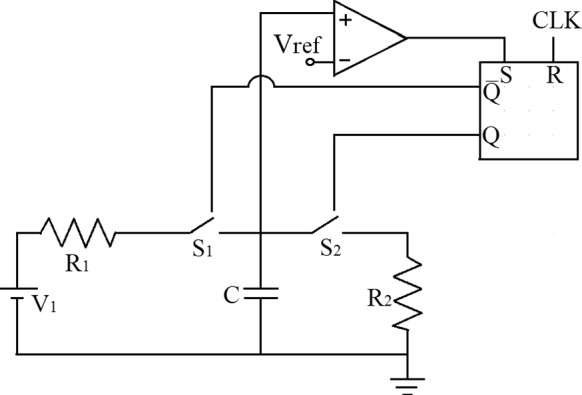
The switching circuit under investigation

Figure 1 illustrates a simple inductorless chaos generator circuit suitable for IC implementation in chaotic communication [36, 40, 41].

The inductorless circuit considered in this paper is a one-dimensional circuit. It consists of a capacitor $\textrm{C}$, which can be charged through a DC input voltage $\mathrm {V_1}$ and resistance $\mathrm {R_1}$ or discharged through a resistance $\mathrm {R_2}$. Two switches, $S_1$ and $S_2$, control the charging and discharging processes, respectively. These switches are controlled by a set-reset (*S*-*R*) flip-flop. A clock signal (CLK) with a period $\textrm{T}$ resets the flip-flop, initiating the charging phase of the capacitor $\textrm{C}$. While the voltage across the capacitor, denoted by $v_C$, is below a reference voltage $\mathrm {V_\textrm{ref}}$, $S_1$ remains ON and $S_2$ OFF. During this period, any arriving clock pulse has no effect. Once the voltage $v_C$ reaches $V_\textrm{ref}$, the latch sets, turning $S_1$ OFF and $S_2$ ON, and the capacitor starts discharging through $R_2$. The arrival of the next clock pulse resets the latch, and the charging mode is turned on again.

The evolution of the state variable $v_C$ from one clock instant to the next can occur in two possible ways. Either $v_C$ reaches the reference voltage before the arrival of the next clock pulse, leading to an ON period followed by an OFF period, or the clock pulse arrives before $v_C$ reaches $V_\textrm{ref}$, resulting in a continuous ON period lasting for $\textrm{T}$ time. The type of evolution that occurs during a specific clock period depends on the rate of charging, which, in turn, depends on the input voltage $V_1$. Thus, $V_1$ is considered a bifurcation parameter in this context. The clock is practically generated by producing a periodic frequency waveform $f = \frac{1}{T}$ with a very low duty cycle. The parameters *C*, $R_1$, $R_2$, $V_\textrm{ref}$, and *f* are kept fixed in the analysis. Assuming all components to be ideal, the governing equations of the system are given by:

1 $$\begin{aligned} \frac{dv_\textrm{C}(t)}{dt} = \left\{ \begin{array}{lrl} \frac{V_1 - v_\textrm{C}(t)}{CR_\textrm{1}}, &{} \text {for }&{} v_\textrm{C}(t) < V_\textrm{ref}\\ -\frac{v_\textrm{C}(t)}{CR_\textrm{2}}, &{} &{} \textrm{otherwise} \end{array}\right. \end{aligned}$$

**Fig. 2:**
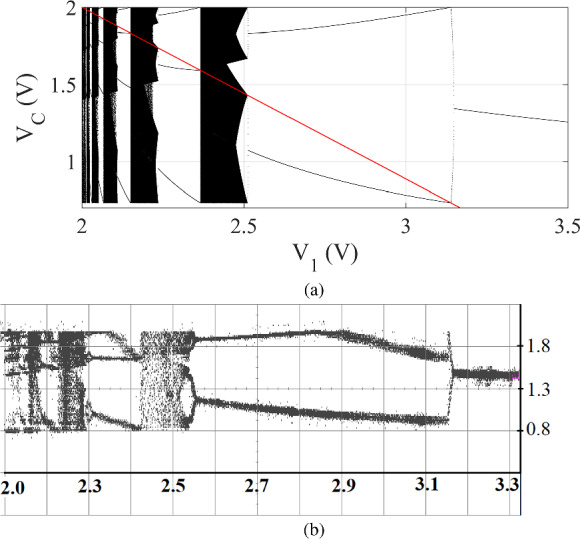
**a** Numerically and **b** Experimentally obtained bifurcation diagrams of the inductorless chaos generator circuit. The horizontal axis is the bifurcation parameter $V_1$ in V, and the vertical axis is the sampled value of the capacitor voltage, $V_\textrm{C}$ in V. The red line in the numerically obtained figure denotes the border voltage, $V_b$, in the parameter space $V_1$. The parameter values are: $R_1 = 9.0~\mathrm {k \Omega }, R_2 = 6.7~\mathrm {k \Omega }, C = 9.9~\textrm{nF}, f = 15~\textrm{kHz}$, and $V_\textrm{ref} = 2.0~\textrm{V}$. (Color online.)

After deriving the stroboscopic map of the system using equation (1) [10], which relates the values of the state variable $v_C(t)$ at a clock instant with that at the previous one, we obtain:

2 $$\begin{aligned} V_{\textrm{Cn}+1}=\left\{ \begin{array}{lll} V_1-\left( V_1-V_{\textrm{Cn}}\right) \cdot e^{-\frac{T}{C R_1}}, &{} \text{ for } &{} V_{\textrm{Cn}} \le V_{\textrm{b}} \\ V_{\textrm{ref}}\left( \frac{V_1-V_{\textrm{Cn}}}{V_1-V_{\text{ ref } }}\right) ^{\frac{R_1}{R_2}} e^{-\frac{T}{C R_2}}, &{} \text{ for } &{} V_{\textrm{Cn}} \ge V_{\textrm{b}} \end{array}\right. \end{aligned}$$

where, $V_\textrm{Cn}$ and $V_\mathrm{Cn+1}$ are the values of the voltage across the capacitor *C* at the *n*-th and $(n+1)$-th clock instants, respectively. The border is defined as $V_{\text {b}} = V_1 - \left( V_1-V_{\text {ref}}\right) e^{\frac{T}{C R_1}}$. The numerically and experimentally obtained bifurcation diagrams are shown in Fig. 2a and b, respectively [10]. The bifurcation parameter is $V_1$, varying between 2.0 V to 3.5 V. The constant parameter values are: $R_1 = 9.0~\mathrm {k \Omega }, R_2 = 6.7~\mathrm {k \Omega }, C = 9.9~\textrm{nF}, f = 15~\textrm{kHz}$, and $V_\textrm{ref} = 2.0~\textrm{V}$. These bifurcation diagrams reveal the period-incriminating cascade phenomena with chaotic windows under the variation of $V_1$.

A detailed investigation of the property of this map, both analytically and numerically, is shown in [9]. This paper shows that the map, for symmetric condition (i.e., $R_1 = R_2$), is reduced to a piecewise-linear map [6]. The map has a unique fixed point that depends on the parameter values $V_1$, $R_1$, $R_2$, and $\textrm{f}$. Under certain conditions, the fixed point undergoes a degenerate flip bifurcation, after which a stable period-2 cycle exists. A detailed experimental investigation of the period-incrementing cascade behavior of this system is shown in [10].

In [37], the dynamics of a stochastic logistic map were shown where the parameter is chosen from a distribution. The logistic map is a smooth map that shows period-doubling bifurcation [42]. There are periodic windows between chaotic attractors in the bifurcation diagram. In contrast, the period-increment cascade bifurcation only occurs in switching dynamical systems under the variation of one of the parameter values [11, 43, 44]. The periodic windows in the bifurcation diagram have finite widths between chaotic attractors. The difference lies in the fact that the periodic windows here have particular identities different from those in the smooth map. Therefore, the paper focuses on the dynamics of the non-smooth system, which generates a period-increment bifurcation. The dynamics of the piecewise-smooth map are studied by introducing stochasticity within the system parameters.

In this paper, we have considered the numerically obtained bifurcation diagram for its convenience in accurately measuring the periodicity of periodic windows and bifurcation points. In contrast, the experimentally obtained diagram suffers limitations. The least count of the scale from the oscilloscope screen is not small enough to measure the exact values of the bifurcation points, as in numerical measurements. Also, the higher periodicities of periodic windows can not be found distinctly because of the inclusion of the circuit noise. The experimentally obtained bifurcation diagram is showcased to verify the different dynamics observed in numerical simulation under the variation of $V_1$ present in the physical system under consideration.

## Methods of introducing stochasticity

In real-life applications, most dynamical phenomena, whether characterized by smooth or piecewise-smooth dynamics, exhibit an inherent stochasticity stemming from randomness in initial conditions of the state variables or fluctuations in parameter values. Additionally, in the case of nonsmooth dynamical systems, stochasticity arises due to fluctuations in border values. In this study, we have investigated the dynamics of  (1) in the presence of stochasticity within the parameter space. Two distinct probability distributions are employed to introduce this stochasticity: uniform and triangular.

To visualize the piecewise-smooth map described in equation (2), sequential observations of the state variable, $v_C$, were conducted, synchronized with the time period of an external clock pulse. The values of the state variable at the *n*-th and $(n+1)$-th periods of the clock signal were recorded as $V_\textrm{Cn}$ and $V_\mathrm{Cn+1}$, respectively. Instead of observing the entire waveform of the state variable, the focus was on discrete points corresponding to the increasing edge of the pulse. The functional form of the map, as shown in equation (2), can be generally represented as

3 $$\begin{aligned} V_\mathrm{Cn+1} = f\left( V_\textrm{Cn}\right) \end{aligned}$$

This general representation of the map (3) is deterministic, which means that at any instant, we can characterize it by fixed parameter values. The DC input voltage, $V_1$, is successively varied for the purpose of observing qualitative change in dynamics. In order to introduce stochasticity into the system as depicted in the case of a smooth map [37], a modification to equation (3) is proposed as follows:

4 $$\begin{aligned} V_\mathrm{Cn+1} = g\left( V_\textrm{Cn},V_1(n)\right) \end{aligned}$$

where,

5 $$\begin{aligned}&g\left( V_\textrm{Cn}, V_1(n)\right) \nonumber \\&=\left\{ \begin{array}{lll} V_1(n)-\left( V_1(n)-V_{\textrm{Cn}}\right) \cdot e^{-\frac{T}{C R_1}}, &{} \text{ for } &{} V_{\textrm{Cn}} \le V_{\textrm{b}} \\ V_{\textrm{ref}}\left( \frac{V_1(n)-V_{\textrm{Cn}}}{V_1(n)-V_{\text{ ref } }}\right) ^{\frac{R_1}{R_2}} e^{-\frac{T}{C R_2}}, &{} \text{ for } &{} V_{\textrm{Cn}} \ge V_{\textrm{b}} \end{array}\right. \end{aligned}$$

From equation (5), we can say that the parameter values at each iteration instant, denoted as $V_1(n)$, are now stochastically selected from a predefined distribution within the bounds of $q_1$ and $q_2$, where *n* is the iteration number. Apart from that, the functional form of the map remains unchanged as described in equation (3). The values of $q_1$ and $q_2$ are the minimum and maximum bifurcation parameter values of the deterministic piecewise-smooth map. In our case, they are $q_1 = 2.0$ V and $q_2 = 3.5$ V. The updated version of the map is considered a *stochastic map*.

In this context, a question arises concerning the potential manifestation of behavior of the deterministic piecewise-smooth map within this stochastic mapping, such as whether there exists any telltale sign of chaos or periodicity similar to the deterministic version of the map. Also, how the dynamics of the deterministic map change when incorporating stochasticity in the parameter space is another important aspect. Finally, there is also a question of whether this stochastic system exhibits ergodicity and, if so, how it distinguishes itself from its deterministic counterpart. To address these inquiries, two methods for computing ensemble averages are adopted, which serve as tools to explore the implications of inherent stochasticity [17, 37, 45]:

- *The Traditional Method (TM)*.
- *The Nature versus Nurture Method (NVN)*.

### TM method

**Table 1:** Tabular representation of TM Method

$V_1(0)$	$V_1(1)$	$V_1(2)$	$V_1(3)$	$V_1(4)$	$V_1(5)$	$V_1(6)$	$V_1(7)$	$V_1(8)$
$V_{C0}$	$V_{C1}$	$V_{C2}$	$V_{C3}$	$V_{C4}$	$V_{C5}$	$V_{C6}$	$V_{C7}$	$V_{C8}$
$\Delta _{t}^0$	$\Delta _{t}^1$	$\Delta _{t}^2$	$\Delta _{t}^3$	$\Delta _{t}^4$	$\Delta _{t}^5$	$\Delta _{t}^6$	$\Delta _{t}^7$	$\Delta _{t}^8$
$V_{C0}^{\prime }$	$V_{C1}^{\prime }$	$V_{C2}^{\prime }$	$V_{C3}^{\prime }$	$V_{C4}^{\prime }$	$V_{C5}^{\prime }$	$V_{C6}^{\prime }$	$V_{C7}^{\prime }$	$V_{C8}^{\prime }$

In this approach, the process is initiated by fixing a small value, denoted as $\Delta _t^0$, which typically is of the order of $10^{-1}$ V, $10^{-2}$ V, and so on. Subsequently, we randomly select the initial value of the state variable $V_C$, along with its perturbed counterpart, denoted as $V_C^{\prime }$. The difference between their values at each iteration, denoted as $\Delta _t^i$, is calculated using the expression $|V_\textrm{Ci}-V_\textrm{Ci}^\mathrm{\prime }|$, where the index ‘*i*’ signifies the *i*-th iteration of state variables for $\Delta _t$ calculation. It is essential to ensure that the initial values of $V_C$ and $V_C^{\prime }$ are chosen within the 0.7 V and 2.0 V range. For instance, suppose we choose $\Delta _t^0 = 10^{-3}$ V and $V_{C0} = 0.2$ V, then $V_{C0}^{\prime }$ would be 0.201 V. If $\Delta _t$ increases over iterations or stabilizes to a finite value other than zero value (since it cannot grow indefinitely because of the bounded behavior of the system), we infer the system has entered a chaotic regime.

Table 1 provides a detailed calculation of $\Delta _t$ at each iteration over eight iterations using the traditional method. The following steps outline the process for these calculations.

1. We first choose an arbitary initial value, $V_\textrm{C0}$, in the range between 0.7 V to 2.0 V and a small value of $\Delta _\textrm{t}$, i.e., $\Delta _t^0$, which makes $V_\textrm{C0}^\prime = V_\textrm{C0} + \Delta _{t}^0$. $V_\textrm{C0}^\prime$ also requires to be within 0.7 V to 2.0 V.
2. We evolve $V_\textrm{C}$ and $V_\textrm{C}^\prime$ independently using the random parameter values of $V_1$ obtained from a distribution ranging between 2.0 V to 3.5 V, up to eight iterations. We then note the values of $V_C$ and $V_\textrm{C}^\prime$ at each iteration. This is shown in Table 1.
3. At each iteration, we calculate $\Delta _{t}^{i}=|V_{Ci}-V^{\prime }_{Ci}|$.
4. To compute the ensemble average of $\Delta _{t}$, we would follow a similar procedure to the one previously described. We begin by selecting another random initial state variable, denoted as $V_\textrm{C0}$, within the interval (0.7 V, 2.0 V), and set $V^{\prime }_\textrm{C0} = V_\textrm{C0} + \Delta _{t}^0$, where, $\Delta _{t}^0$ remains consistent with the initial separation used previously. For this procedure, we also have to randomly choose the bifurcation parameter $V_1$ from the distribution ranging from 2.0 V to 3.5 V.

For each iteration indexed by *i*, the state variable $\Delta _{t}^i$ is computed individually for every $V_\textrm{C0}$, and the corresponding average is determined by aggregating all $\Delta _{t}^i$ values at that particular iteration. This iterative process is repeated across 10000 initial values for $V_\textrm{C0}$. Throughout these iterations, $\Delta _{t}^0$ remains fixed initially.

After computing and averaging the $\Delta _{t}^i$ values, denoted as $\langle \Delta _\textrm{t}\rangle$, these resultant average values are plotted against the iteration number, *n*. This graphical representation enables us to observe and track the progression of $\Delta _{t}$ over the course of iterations, providing insights into its behavior and dynamics within the system.

### NVN method

This method initially eliminates the need for selecting a fixed value for $\Delta _{t}^0$. Instead, we start the process by randomly choosing an initial value for the state variable $V_C$, denoted as $V_\textrm{C0}$, within the interval (0.7 V, 2.0 V). Subsequently, we iterate the system using a set of randomly selected bifurcation parameter values $V_1$ drawn from a specific distribution. To compute $\Delta _{t}$ at each iteration, we commence with the same initial value $V_C$ but evolve it using another set of randomly selected bifurcation parameters, denoted as $V^{\prime }_1$, obtained from the same distribution. Therefore, we set $\Delta _{t}^0 = 0$ V. To calculate the ensemble average of $\Delta _t$, we repeat the same process for different $V_\textrm{C0}$ values and compute the average of $\Delta _t$ at each iteration. Table 2 illustrates the tabular representation of $\Delta _t$ over eight iterations.

**Table 2:** Tabular representation of NVN Method

$V_1(0)$	$V_1(1)$	$V_1(3)$	$V_1(3)$	$V_1(4)$	$V_1(5)$	$V_1(6)$	$V_1(7)$	$V_1(8)$
$V_{C0}$	$V_{C1}$	$V_{C2}$	$V_{C3}$	$V_{C4}$	$V_{C5}$	$V_{C6}$	$V_{C7}$	$V_{C8}$
$V^{\prime }_1(0)$	$V^{\prime }_1(1)$	$V^{\prime }_1(2)$	$V^{\prime }_1(3)$	$V^{\prime }_1(4)$	$V^{\prime }_1(5)$	$V^{\prime }_1(6)$	$V^{\prime }_1(7)$	$V^{\prime }_1(8)$
$V_\textrm{C0}$	$V_\textrm{C1}^{\prime }$	$V_\textrm{C2}^{\prime }$	$V_\textrm{C3}^{\prime }$	$V_\textrm{C4}^{\prime }$	$V_\textrm{C5}^{\prime }$	$V_\textrm{C6}^{\prime }$	$V_{C7}^{\prime }$	$V_{C8}^{\prime }$
$\Delta _{t}^0$	$\Delta _{t}^1$	$\Delta _{t}^2$	$\Delta _{t}^3$	$\Delta _{t}^4$	$\Delta _{t}^5$	$\Delta _{t}^6$	$\Delta _{t}^7$	$\Delta _{t}^8$

Next, we explain step-by-step how to obtain the ensemble average of $\Delta _t$ over eight iterations.

1. We begin by defining $V_\textrm{C0}$ as a randomly chosen value between 0.7 V and 2.0 V, say, 0.9 V. The parameter $V_1$ is then selected from a uniform distribution within the interval between 2.0 V and 3.5 V, producing a set of values such as $\{2.1, 2.7, 2.9,...\}$ volts. Subsequently, we proceed with iterating the state variable and recording the values of the state variable at each iteration. In our work, we have iterated for 10000 values.
2. To compute the value of $\Delta _t$, we initiate a new iteration process using a fresh set of bifurcation parameter values drawn from the identical distribution while maintaining $V_\textrm{C0}$ at 0.9 V as in the previous iteration. This new set of bifurcation parameter values is named as $V_\textrm{1}^\prime$. The values of the state variable in this new iteration are denoted as $V^{\prime }_\textrm{C}$. In each iteration, we calculate $\Delta _{t}$ as the absolute difference between $V_\textrm{C}$ and $V^{\prime }_\textrm{C}$, where $\Delta _{t}^0$ is initialized to 0 V.
3. To calculate the ensemble average of $\Delta _t$, we have repeated the previously described process with a new random initial value for $V_C$, chosen uniformly within the range (0.7 V, 2.0 V). We have repeated the process described in steps 1 and 2 to get a different time series of $\Delta _t$.
4. We iterate the step 3 for different $V_C$ values and compute $\Delta _t$ for each $V_C$ value. Every time, $V_\textrm{CO}$ will be different.
5. Once we obtain $\Delta _t$ values with respect to the number of iterations for various initial values of $V_C$, we calculate the average of $\Delta _t$ for each iteration. This procedure is replicated in our study using 10000 random initial values of $V_C$ within the range between 0.7 V to 2.0 V. Subsequently, by computing the average of $\Delta _{t}$ for each particular iteration, we generate a plot depicting these ensemble averages $\langle \Delta _\textrm{t}\rangle$ against the number of iterations. This analysis allows us to evaluate the convergence behavior of $\Delta _{t}$ with respect to the iterations.

## Ergodicity and convergence

According to Boltzmann’s ergodic hypothesis [46], given any arbitrary initial condition, the state variable under consideration traverses through all accessible points in phase space over an extended period. In the discrete map represented by equation (3), the state variable exhibits maximum oscillation within the range of 0.7 V to 2.0 V. It can be observed from the vertical axis of the bifurcation diagram in Fig. 2a). However, the evolution of the state variable of the deterministic map is not ergodic due to the presence of periodic and chaotic attractors under the parameter variation, eventually causing it to converge to equilibrium points. The effects of stochasticity in parameter space are compared to the deterministic map described by equation (5) to investigate the ergodic nature of this stochastic state variable contingent upon distributions with finite endpoints.

While varying the bifurcation parameter $V_1$ in the forward direction, the deterministic map exhibits a reverse period-incrementing cascade phenomenon with intermittent chaotic windows between periodic attractors, which implies that while $V_1$ decreases smoothly, $V_C$ displays an interplay between periodic and chaotic windows. The map possesses the border value in every parameter configuration, where the next iteration will land on the switching voltage value at 2.0 V. The red line in Fig. 2a is the border under the variation of the parameter $\mathrm {V_1}$.

**Fig. 3:**
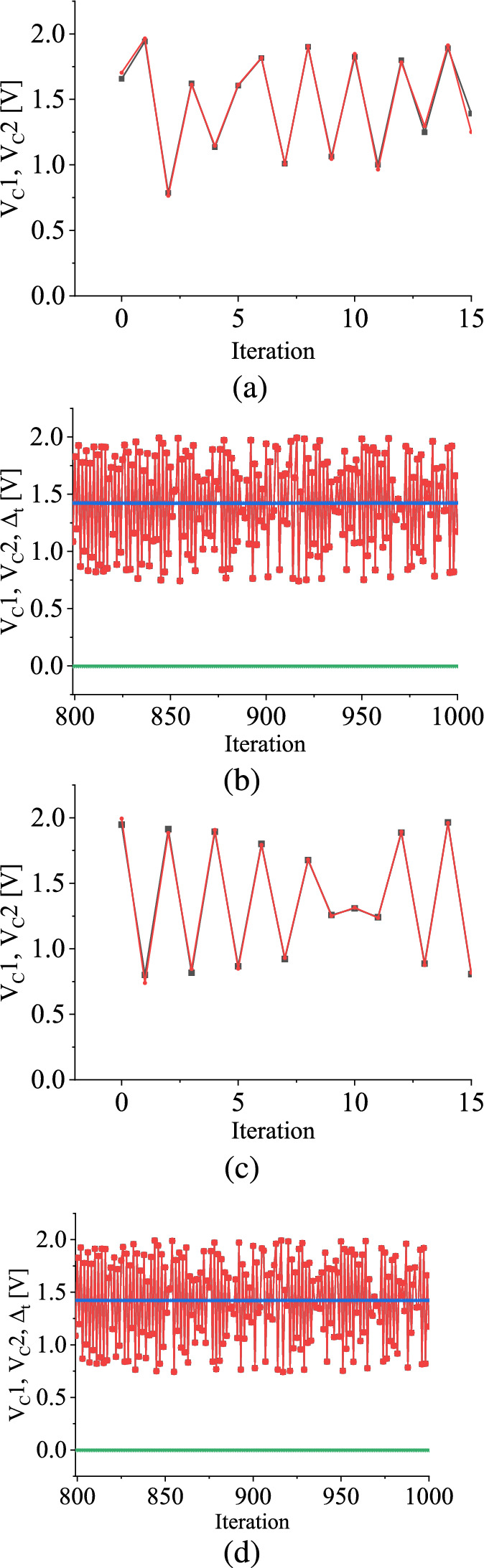
TM method results: The evolutions of $V_C1$ (in red color) and $V_C2$ (in black color), $\overline{V_\textrm{C}}$ (in blue color) in V, and $\mathrm {\Delta _t}$ (in green color) in V with the number of iteration *n* in the case of **b** uniform and **d** symmetric triangular distributions ranging between 2.0 V to 3.5 V. **a** and **c** are the zoomed time series of $V_C1$ and $V_C2$. The initial separation, $\mathrm {\Delta _{t}^0} = 0.1$ V. (Color Online.)

In contrast, in the case of the stochastic map, where randomness is introduced in the parameter space having the initial value of the state variable $V_C$ randomly chosen between 0.7 V and 2.0 V, $V_C$ exhibits an ergodic behavior, implying that it does not converge to a fixed point (as illustrated in Fig. 3). Consequently, it becomes impossible to predict the *n*-th iteration value of the state variable of the map, $V_\textrm{Cn}$, before the *n*-th iteration. This starkly contrasts the behavior exhibited by the deterministic map, wherein the *n*-th iteration, $V_\textrm{Cn}$, can be readily computed from the functional form of the piecewise-smooth map when the state variable shows deterministic behavior.

An intriguing observation is that when the state variable $V_\textrm{C}1$ and its perturbation $V_\textrm{C}2$ are evolved with a set of bifurcation parameters chosen from a particular distribution at each iteration, they may eventually converge with each other after a certain number of iterations (for large value of *n*). In other words, defining $|V_\textrm{C}1 - V_\textrm{C}2| = \Delta _\textrm{t}$, after the *n*-th iteration (in our work, $n = 10000$), $\mathrm {\Delta _{t}}$ approaches 0 V. This phenomenon shows the convergence behavior of the two variables despite the random variations introduced through different bifurcation parameters.

Figure 3a and c display zoomed-in views of the evaluations of the state variable and its perturbation values, $V_C1$ and $V_C2$, plotted against the iteration number for uniform and symmetric triangular distributions, respectively. These figures illustrate that the waveforms of $V_C1$ and $V_C2$ exhibit irregular behaviors and do not converge to fixed points. However, after a certain number of iterations, they eventually merge, resulting in $\Delta _t$ becoming zero. This merging process is depicted in Fig. 3b and d, respectively. As a result of this merger, $V_C1$ and $V_C2$ can no longer be observed separately.

Next, the discrete-time average of $V_\textrm{C}$, denoted as $\overline{V_\textrm{C}}$, is calculated considering the number of iterations. Figure 3b and d illustrate that $\overline{V_\textrm{C}}$ converges to a fixed value when the bifurcation parameter $V_1$ is drawn from the uniform distribution and the symmetric triangular distribution. The fixed values of $\overline{V_\textrm{C}}$ for the uniform and the symmetric triangular distributions are 1.423 V and 1.415 V, respectively.

Since $V_\textrm{C}$ exhibits an ergodic behavior, we can assume that the state variable possesses $V_\textrm{Cmax}$ and $V_\textrm{Cmin}$ values in the discrete-time series waveforms. We denote the difference between these values as $\Delta _\textrm{V} = (V_\textrm{Cmax} - V_\textrm{Cmin})$ in the parameter space. Our goal is to examine how $\Delta _{V}$ varies concerning the change in the range of the distribution of bifurcation parameters. This analysis thus determines whether this stochastic map effectively covers all regions in the phase space for a given choice of distribution.

**Fig. 4:**
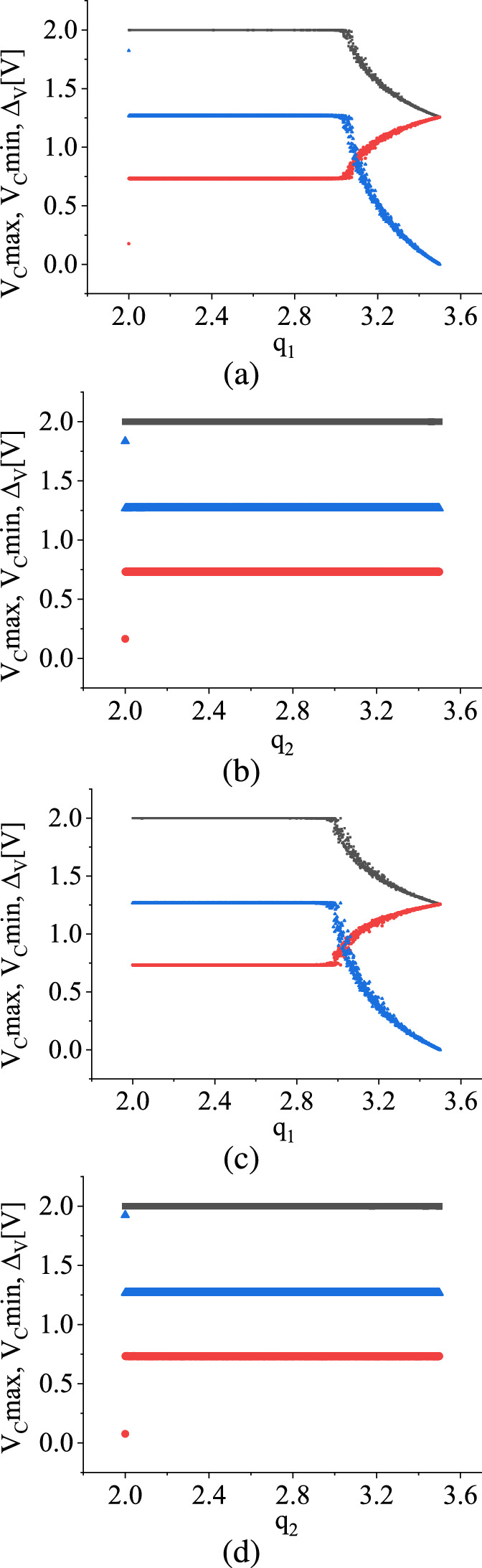
TM method results: **a** and **b** show the variations of $V_\textrm{Cmax}$ (black in color), $V_\textrm{Cmin}$ (red in color), and $\Delta _\textrm{V}$ (blue color) for uniform distribution and **c** and (d) show the variations of $V_\textrm{C}max$, $V_\textrm{C}min$ and $\Delta _\textrm{V}$ for the symmetric triangular distributions. In the case of **a** and **c**, $q_1$ is varied from 2.0 V to 3.5 V while $q_2$ is fixed at 3.5 V, and in the case of **b** and **d**, $q_2$ is varied from 2.0 V to 3.5 V while $q_1$ is fixed at 2.0 V. (Color online.)

A uniform distribution between the range $q_1$ and $q_2$ for the bifurcation parameter $V_1$ is considered, where $q_1$ and $q_2$ denote the minimum and maximum values of the range of distribution, respectively. Subsequently, $q_2$ is fixed at 3.5 V while $q_1$ is varied in the range 2.0 V to 3.5 V. This generated Fig. 4a and b where ergodicity of the variable $V_\textrm{C}$ for uniform distribution, and Fig. 4c and d for symmetric triangular distributions are observed.

From Fig. 4a, it is evident that the ergodicity is consistent up to $q_1 = 3.04$ V, but beyond that point, it breaks, and the variable converges to a value of 1.25 V. This value of $q_1$, where $\Delta _\textrm{V}$ changes, closely corresponds to the bifurcation parameter value at which a period-2 orbit undergoes a border collision bifurcation and transforms into a period-2 orbit ($V_1 \approx 3.1~\textrm{V}$) for the deterministic map. The nature of ergodicity for the symmetric triangular distribution under the same condition is observed next in Fig. 4c. Notably, the behavior of the ergodicity with the variation of $q_1$ is similar to that of the uniform distribution. Here, the ergodicity breaks at around $q_1 \approx 3.0$ V.

Finally, the ergodic behavior by fixing $q_1$ at 2.0 V and varying $q_2$ for the uniform distribution and symmetric triangular distribution, as depicted in Fig. 4b and d, respectively, are investigated. In both cases, the ergodicity persists throughout the parameter space with the variation of $q_2$, while $q_1$ remains fixed at 2.0 V. This observation implies that there exists a finite likelihood of encountering the state variable $V_C$ within the entirety of the phase space of the map, where the distribution spans the interval between $q_1$ and $q_2$.

## Invariant probability density function

In [38], it was demonstrated that the distribution of the state variable converges to a Maxwell-Boltzmann distribution in the limit of a long time for a stochastic map. In contrast, the distribution observed in our specific stochastic map closely resembles the shape of the invariant measure associated with a logistic map [39].

**Fig. 5:**
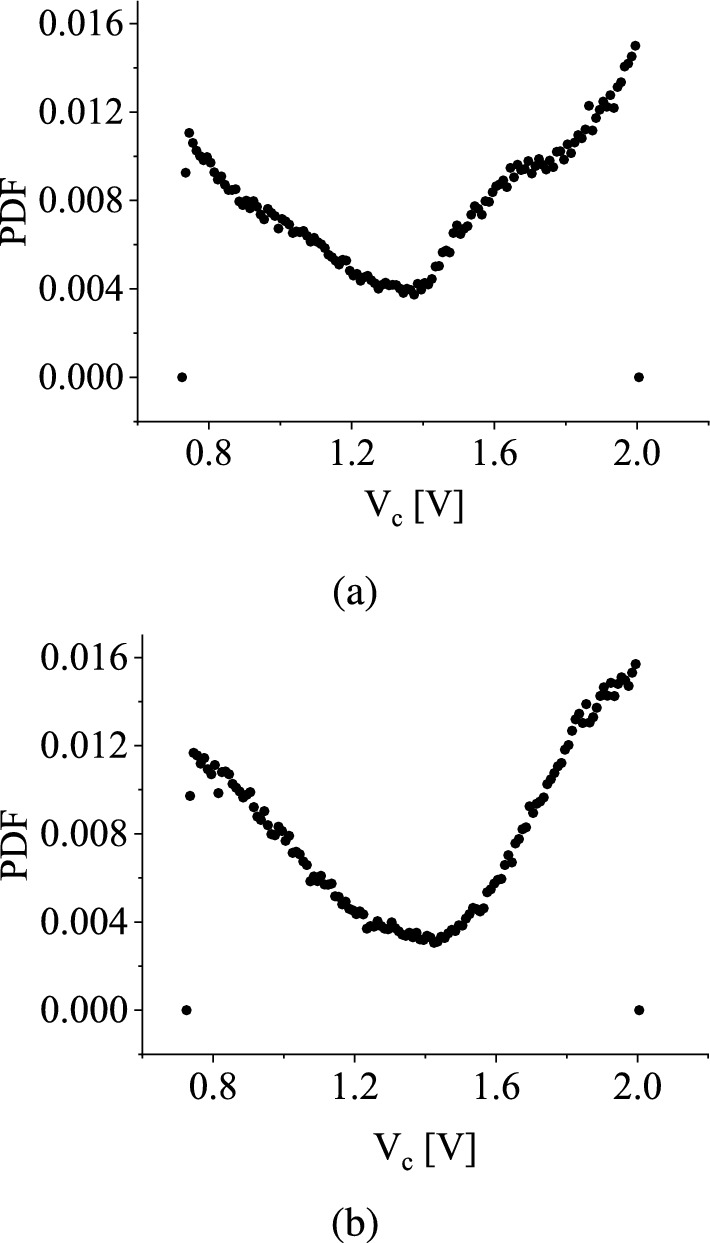
The probability density functions of the state variables **a** for uniform distribution and **b** for symmetric triangular distribution. The lower and upper ranges of the distribution are considered 2.0 V and 3.5 V, respectively, for both distributions

Using uniform distribution and symmetric triangular distribution within 2.0 V and 3.5 V, the probability density function (PDF) of the state variable of the map is plotted, as shown in Fig. 5a and b, respectively. As we have estimated this probability density function numerically, we have plotted the normalized histogram here. The bin center refers to the center of the bin of the histogram in the case of the state variable $V_C$. Notably, these PDFs remain invariant regardless of the initial conditions when the bifurcation parameter is drawn from a fixed distribution with predefined boundary points between 2.0 V to 3.5 V.

Figure 5a and b demonstrate the stochastic behavior of the system for both distributions. As we observe the values along the horizontal axis, we notice that the distribution function progressively decreases until reaching a certain point and then begins to increase. This trend indicates that the random state variable is more likely to be situated in the vicinity of the switching surface at 2.0 V, and the probability of getting the state variable of the function is maximum there. The dips in both curves signify that the probability density function is relatively lower, around 1.5 V, and the probability of obtaining the state variable of the function is minimal there.

It is numerically observed that as long-run iteration progresses, the distribution of $p_t(V_C)$ (which represents the probability density function of $V_C$ at discrete-time *t*) ceases to evolve with time and instead converges towards a fixed distribution denoted as $p(V_C)$. In analogy with the Frobenius-Perron operator used for any deterministic map, we can define a similar operator to derive an invariant measure for this stochastic map.

The expression for the stationary distribution *p*(*x*) as *n* tends to infinity is given by:

6 $$\begin{aligned} p(x) {\mathop {=}\limits ^{n\rightarrow \infty }} \int dy \int _{q_1}^{q_2} dV_1 p(y) \delta (x - g(y,V_1)) \end{aligned}$$

where $x = V_\mathrm{Cn+1}$ and $y = V_\textrm{Cn}$. Although $n\rightarrow \infty$, $p(V_\mathrm{Cn+1}) \approx p(V_\textrm{Cn})$, we can put *p*(*x*) in place of *p*(*y*) in the above equation, to find the *p*(*x*).

In Equation (6), we can observe that the stationary distribution *p*(*x*) is dependent on the parameters $q_1$ and $q_2$, representing the endpoints of the probability distribution, as well as the specific type of probability distribution employed. This dependence has been further corroborated through numerical investigations.

## Evolution of stochastic map

Upon scrutinizing the dynamics of this stochastic map, it has come to light that there exist two distinct regimes of evolution:

- *Non-Chaotic Regime*
- *Chaotic Regime*

Given the stochastic nature of this map, it is impossible to predict the *n*-th iteration value of the state variable $V_\textrm{C}$ prior to the *n*-th iteration. Consequently, the analytic computation of fixed points and their corresponding stability, contingent on this stochastic nature, remains an infeasible task. In light of this, we have undertaken numerical computations for two distinct evolutions of state variables within the stochastic map. These computations involve selecting different initial conditions while maintaining a consistent set of parameter values derived from the distribution for every iteration (referred to as the TM method) or, alternatively, maintaining the same initial condition while selecting a distinct set of parameter values obtained from a distribution (referred to as the NVN method).

Should the difference between the two state variables ($\mathrm {\Delta _t}$) approach zero asymptotically, or should the two evolutions converge to a fixed value so that their difference will be zero in later iterations, this scenario is designated as a *periodic* or *non-chaotic regime*. It is worth noting that for the prediction of periodicity, we must observe a minimum of two distinct evolutions. Conversely, suppose the separation between the two state variables ($\Delta _t$) does not asymptotically approach zero but instead attains a fixed nonzero value. In that case, this scenario is classified as a *chaotic regime*, as the two evolutions ultimately fail to converge in subsequent iterations [17].

### Results for TM method

#### Non-chaotic regime

In Fig. 3, we show a pair of initial values of $V_C$, which are slightly different and allow them to evolve as a function of time within the parameter range between 2.0 V and 3.5 V. We observe that $V_C$ does not attain a fixed point value. However, when the separation, $\mathrm {\Delta _t}$, between two state variables gradually decays towards zero after certain iterations, the system approaches ordered behavior. The region in parameter space where this condition occurs is called a *regular or non-chaotic regime*. When $\mathrm {\Delta _t}$ is zero, we can say there is no damage to the state variable of the stochastic map [37].

**Fig. 6:**
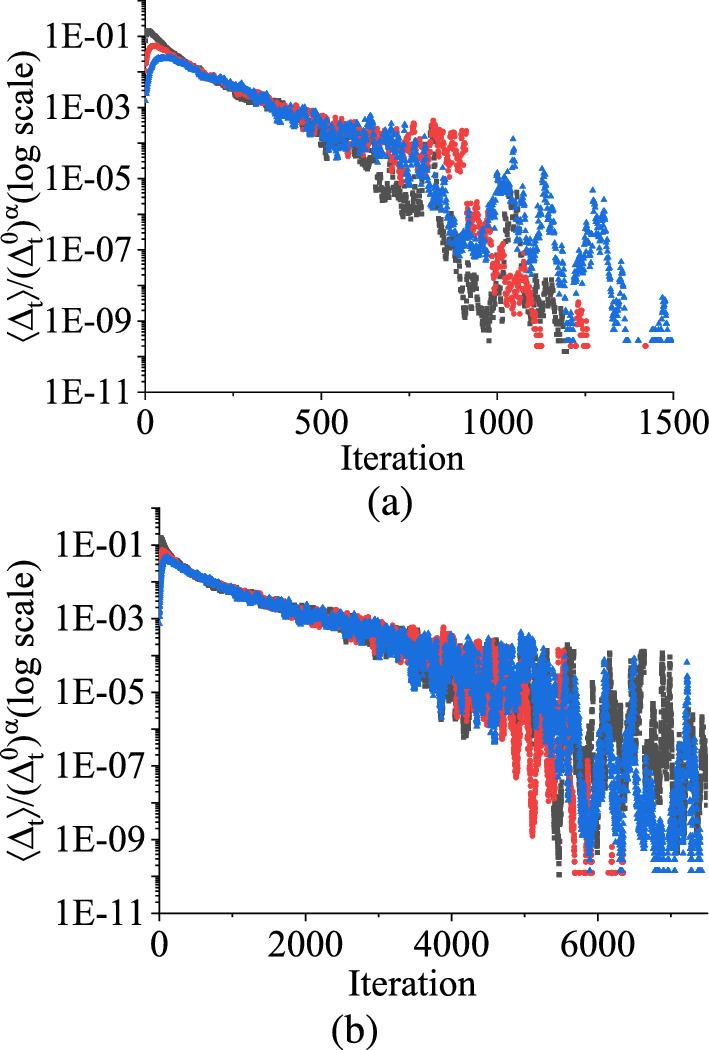
TM method results: The scaled value of damage $\Delta _{t}$ against the number of iteration *n* in the case of **a** uniform and **b** symmetric triangular distributions with a range of 2.0 V to 3.5 V. The black color is for the initial separation value between two variables, $\Delta _t^0$ is 0.1 V, the red curve is for $\Delta _t^0 = 0.01$ V, and the blue curve is for $\Delta _t^0 = 0.001$ V. (Color online.)

In a non-chaotic regime, when starting from an initial random value of $\Delta _t^0$ and measuring the difference $\Delta _\textrm{t}$ between two independently chosen variables $V_\textrm{C}1$ and $V_\textrm{C}2$ after a certain number of iterations, it eventually converges to zero. To quantify this behavior, we numerically demonstrate the scaled value of ensemble averages of $\Delta _\textrm{t}$, representing the amount of convergence with the number of iterations using the TM method, as shown in Fig. 6. Figure 6a illustrates the variation of damage with the number of iterations for the uniform distribution, and Fig. 6b depicts the same for symmetric triangular distribution.

The two figures of the scaled value of damage in Fig. 6 are plotted using the following steps:

1. We compute the ensemble average of $\Delta _t$ following the methodology outlined in Sect. 3.1 for the TM method.
2. By choosing $\Delta _t^0$ to different values, we generate distinct time series of $\Delta _t$ relative to the number of iterations.
3. We generate the values of the ensemble averages of $\Delta _t$ in the log scale with the number of iterations for different initial separations $\Delta _t^0$. Employing the data collapsing technique, we ascertain the scaling exponent of $\Delta _t^0$.
4. Finally, we create the log-log plot of normalized ensemble average values of $\Delta _t$ relative to $\Delta _t^0$, $\left\{ \langle \Delta _t\rangle / (\Delta _t^0)^\alpha \right\}$ for various iterations.

In both cases depicted in Fig. 6, we examine the behavior of the scaled value of the damage for three initial random numbers of $\Delta _\textrm{t}$, such as 0.1 V, 0.01 V, and 0.001 V. Initially, the three different initial scaled values of $\Delta _t$ increase up to a certain number of iterations, and then they start to decrease as the iterations progress. Eventually, these scaled values of $\Delta _\textrm{t}$ merge together, exhibiting a convergence towards a common trajectory. After some iterations, the scaled value of $\Delta _\textrm{t}$ demonstrates oscillatory behavior in each figure. Despite this oscillation, it is important to note that $\Delta _t$ in each case converges towards zero as the iteration number *n* progresses. However, the convergence rate of $\langle \Delta _t\rangle$ is faster for the uniform distribution than the symmetric triangular distribution, as shown in Fig. 6. The two figures in Fig. 6 show that although the state variable $V_C$ does not attain a periodic behavior within the parameter range in between 2.0 V and 3.5 V, the separation $\Delta _t$ between the state variable and its perturbation values for three different $\Delta _t^0$ values decay towards zero as time progresses. As mentioned earlier, it shows the ordered behavior (as the state variable shows the periodic) of the stochastic map in the parameter range. As each plot in Fig. 6 demonstrates that the time-averaged scaled value of damage, denoted by $\langle \Delta _\textrm{t}\rangle$, exhibits monotonically decreasing value towards zero for both distributions, even though they have the same distribution range, this behavior suggests a regular or non-chaotic nature in the system.

Furthermore, the relationship between $\Delta _\textrm{t}$ and $\Delta _t^0$ is nonlinear, specifically given by $\langle \Delta _\textrm{t}\rangle \propto ({\Delta _t^0})^\alpha \exp (\lambda n)$, where $\Delta _t^0$ is the initial separation between the initial values of the state variable, $V_\textrm{C}$. In contrast, the deterministic version of this map follows $\Delta _\textrm{t} \propto \Delta _t^0\exp (\lambda n)$. *n* is the discrete-time representation here, which means the iteration values. The exponent $\alpha$ is estimated to be $0.15\pm 0.01$ for the uniform distribution and $0.05\pm 0.01$ for the symmetric triangular distribution. The Lyapunov exponent ($\lambda$) exhibits a pronounced sensitivity to the endpoints, $q_1$ and $q_2$, of the distributions, which decreases as $q_2 - q_1$ is decreased, whereas for the smooth map, the $\lambda$ increases [17]. The corresponding Lyapunov exponents are approximately $\lambda \simeq -5 \times 10^{-3}$ for the uniform distribution and $\lambda \simeq -1.64 \times 10^{-3}$ for the symmetric triangular distribution having the range in between 2.0 V to 3.5 V. The approximated Lyapunov exponents represent the separation rates of initially close trajectories in phase space. A more negative Lyapunov exponent signifies a faster convergence of nearby trajectories, indicating greater stability in the system.

These findings provide quantitative estimates and insights into the relationship between the initial scaled damage $\Delta _t^0$ and its subsequent evolution over time, characterized by the time-averaged scaled damage $\langle \Delta _\textrm{t}\rangle$ for the considered stochastic piecewise-smooth map. The observed nonlinear relationship with the exponent $\alpha$ indicates the sensitivity of the response of the system to the initial conditions, especially when considering random distributions.

**Fig. 7:**
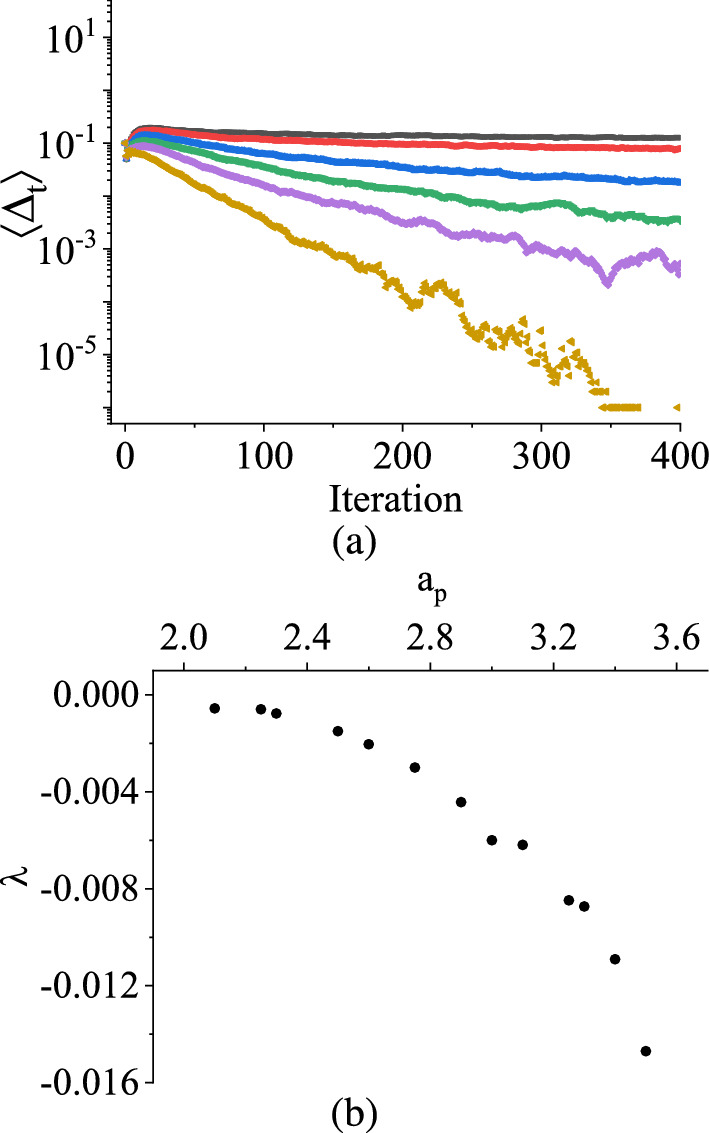
TM method results: $\langle \Delta _{t}\rangle$ in V against the number of iteration n for the asymmetric triangular distribution with different peaks at $a_p$ in V. Black color is for $a_p = 2.25$ V, Red color is for $a_p = 2.5$ V, Blue color is for $a_p = 2.75$ V, Green color is for $a_p = 3.0$ V, Purple color is for $a_p = 3.25$ V, Yellow color is for $a_p = 3.5$ V. **b** the variation of the Lyapunov exponent with the values of $a_p$ in V for the asymmetric triangular distribution peaked at $a_p$ in the range between 2.0 V to 3.5 V (Color Online.)

In Fig. 7a, the variation of $\langle \Delta _t\rangle$ with the number of iterations is presented for asymmetric triangular distributions, where the distribution ranges from 2.0 V to 3.5 V. Six different peaks ($a_p$) were considered to quantify the asymmetries, specifically $2.25~V \le a_p \le 3.5$ V. Notably, it is observed that as $a_p$ increases, the negative slope of $\langle \Delta _\textrm{t}\rangle$ with the number of iterations also increases, indicating a more rapid convergence of $\langle \Delta _\textrm{t}\rangle$ towards zero.

Figure 7b shows the relationship between the Lyapunov exponent ($\lambda$) and $a_p$ using the same algorithm used in [37]. The plot reveals that as $a_p$ progressively increases, the modulus of $\lambda$ also increases exponentially. The interpretation of $\lambda$ suggests that as the value of $a_p$ decreases, $\langle \Delta _\textrm{t}\rangle$ vanishes more gradually. Conversely, as $a_p$ increases, $\langle \Delta _\textrm{t}\rangle$ becomes more prominent, signifying a higher degree of sensitivity to initial conditions and potentially less stable behavior.

**Fig. 8:**
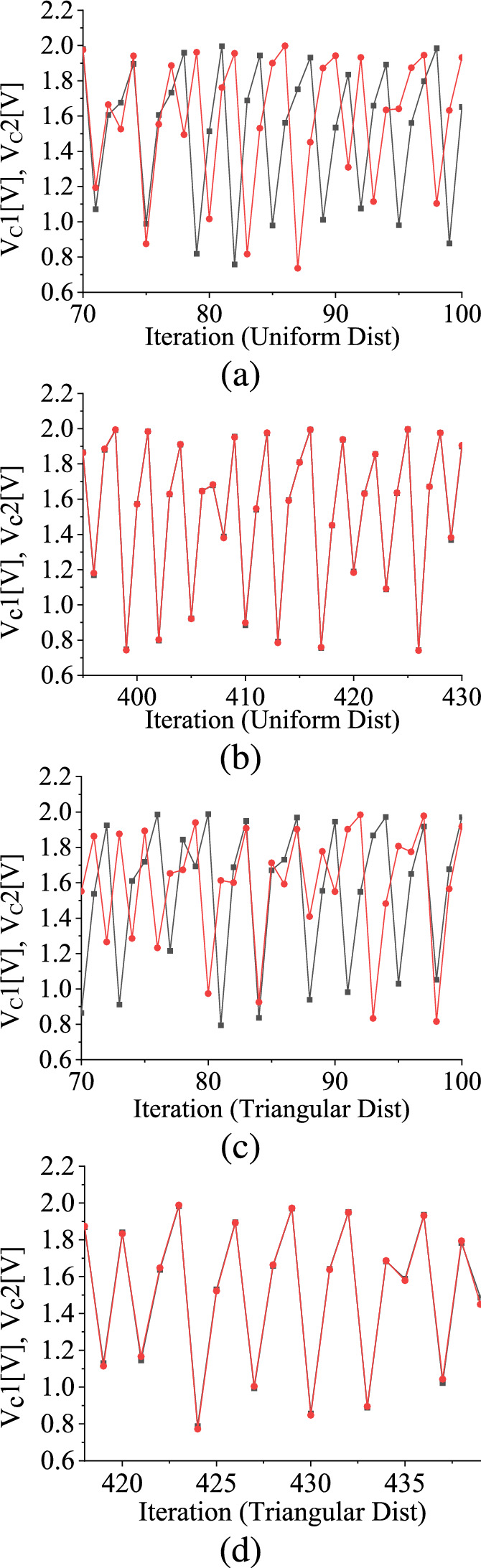
TM method results: Uniform Distribution: Two different evolutions of $V_\textrm{C}$, when **a** $\Delta _{t}$ is nonzero, **b** when $\Delta _{t}$ is zero. Triangular Distribution: Two different evolutions of $V_\textrm{C}$ when **c** $\Delta _{t}$ is nonzero, **d** when $\Delta _{t}$ is zero. Black and red colors are for $V_\textrm{C}1$ and $V_\textrm{C}2$, respectively. The distribution is within the range of 2.0 V and 2.53 V. (Color online.)

#### Chaotic regime

In the previous case, under the non-chaotic regime, we observed that $\langle \Delta _{t}\rangle$ approaches zero with increasing iterations for specific values of $q_1 = 2.0$ V and $q_2 = 3.5$ V. However, when we vary $q_2$ while keeping $q_1$ fixed at 2.0 V, certain scenarios arise where $\langle \Delta _{t}\rangle$ does not reach zero. Instead, it asymptotically converges to a constant nonzero value. This persistent behavior of ergodicity throughout the parameter space, with the system settling into an asymptotic non-zero value of $\langle \Delta _\textrm{t}\rangle$, is defined as *chaotic regime*. We denote the asymptotic non-zero value of $\langle \Delta _\textrm{t}\rangle$ as the saturation of $\Delta _t$, represented as $\Delta _\textrm{sat}$.

**Fig. 9:**
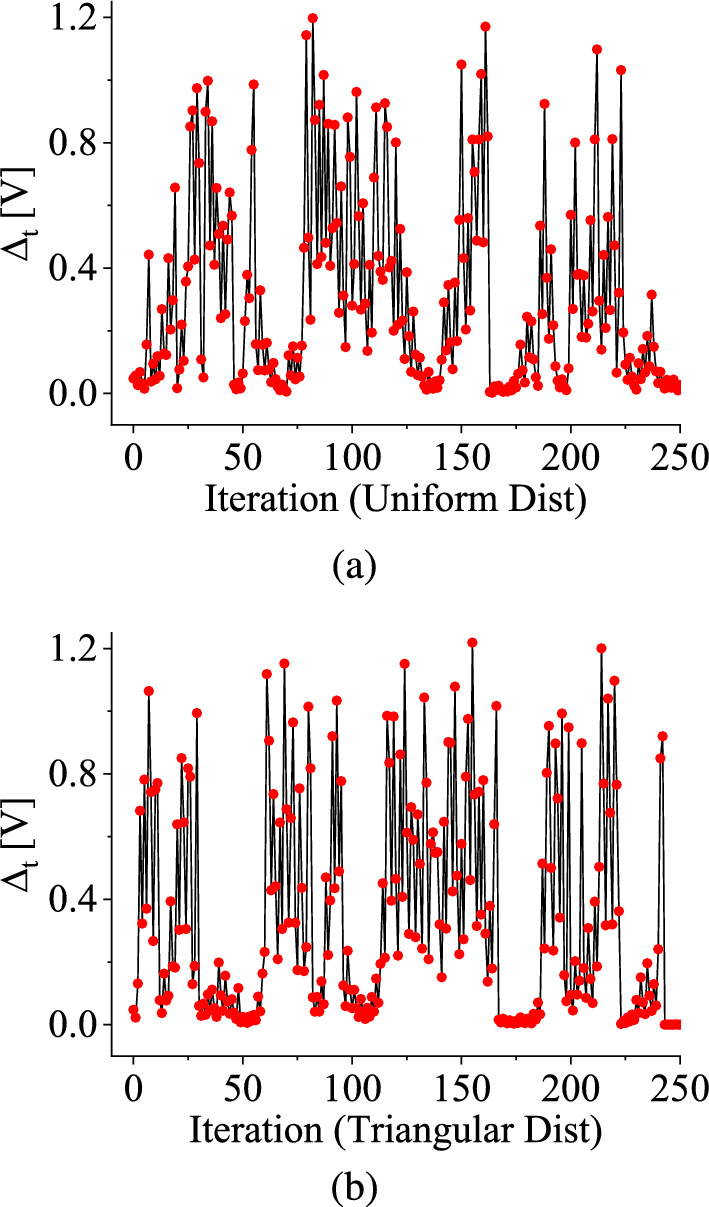
TM method results: The variations of $\Delta _t$ with the number of iteration n for the **a** uniform and **b** triangular distributions. The distribution is within the range of 2.0 V and 2.53 V. (Color online.)

For this specific stochastic map, when a pair of variables $V_\textrm{C}1$ and $V_\textrm{C}2$ are chosen within the chaotic regime, their differences, $\Delta _{t}$, toggles between zero and non-zero values with the iterations when the distribution range is within the interval 2.0 V to 2.53 V as shown in Fig. 8 and Fig. 9, respectively. In Fig. 8a and c, we illustrate the nonzero values of $\Delta _t$ for both uniform and symmetric triangular distributions, respectively. Figure 8b and d are the time series waveforms of two different initial values of the state variable when $\Delta _t$ merges. The time-series waveforms of the state variable and its perturbation, denoted by red and black colors, erratically merge and separate as time progresses. Figure 9a and b show the interplay between zero of non-zero values of $\Delta _t$ as the number of iterations progresses for uniform and symmetric triangular distributions, respectively. The red dots in Fig. 9a and Fig. 9b are the values of $\Delta _t$ for each iteration. The black color denotes the lines joining the red dots.

Initially, $\Delta _{t}$ is zero, indicating no ergodicity for a certain period. Consequently, the waveforms $V_\textrm{C}1$ and $V_\textrm{C}2$ effectively merge as time progresses. However, after this initial phase, the damage $\Delta _t$ assumes a specific non-zero value, causing the two variables to diverge from each other. This separation persists for a particular duration until $\Delta _{t}$ decreases back to zero again, and the waveforms merge. After some iteration, $\Delta _t$ has some non-zero value once more. This cyclic behavior repeats itself as the iterations progress forward. This behavior pattern suggests a dynamic interplay within the chaotic regime involving the amalgamation and division of two state variables over time. This pattern emerges due to the non-smoothness property, i.e., the period-incrementing cascade phenomenon, where there are periodic windows having finite widths in between chaotic attractors in the bifurcation diagram in the case of the deterministic piecewise-smooth map. This particular behavior has not been previously observed in the context of a stochastic smooth map and arises solely as a consequence of the non-smooth behavior of the map [8, 11, 44].

It is worth noting that in the case of the deterministic map, which exhibits a reverse period-incrementing cascade phenomenon with chaotic windows between periodic orbits (as depicted in Fig. 2a), the ratios of the widths of successive periodic windows and subsequent chaotic windows converge to a constant value [10]. However, in contrast, for the stochastic map, the ratios of consecutive spans where $\Delta _{t}$ is zero and the ratios of successive spans with nonzero $\Delta _t$ are random and do not converge to a fixed value.

The above-mentioned findings emphasize the distinct and complex nature of the behavior of the stochastic piecewise-smooth map in the chaotic regime, which never occurs in a smooth map, showcasing the significance of randomness in shaping its dynamics and ergodic properties.

**Fig. 10:**
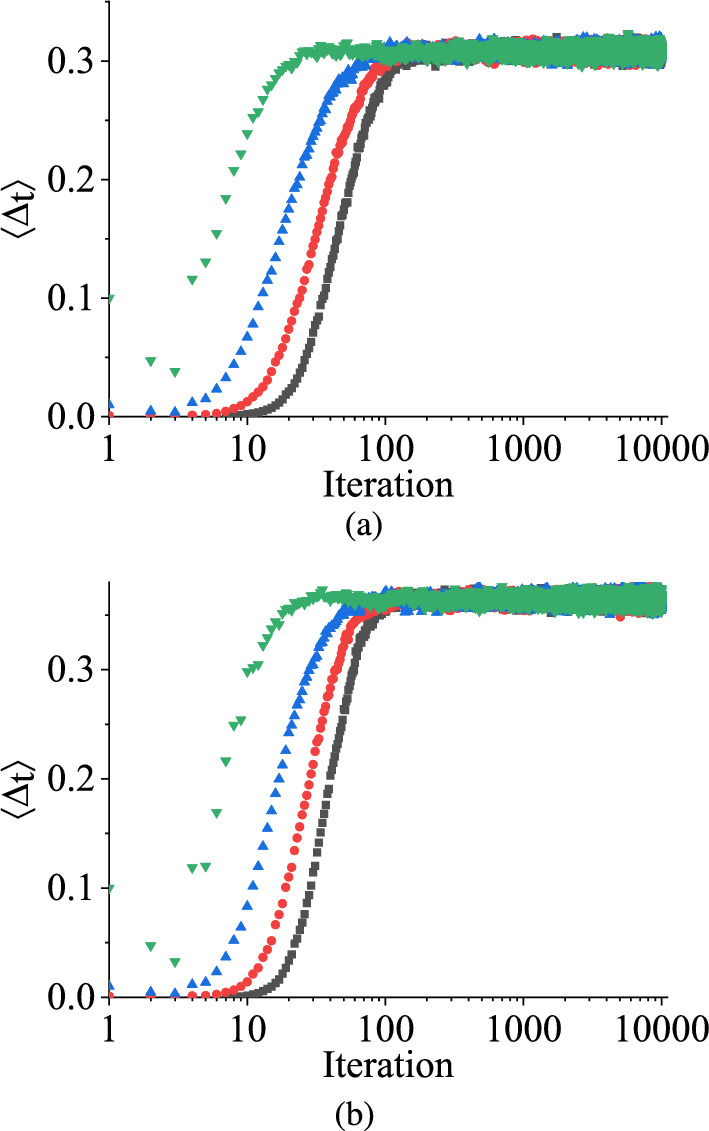
TM method results: $\langle \Delta _{t}\rangle$ in V against the number of iteration n for **a** uniform distribution and **b** symmetric triangular distribution having the range between $q_1 = 2.0$ V and $q_2 = 2.53$ V. For black, red, blue, and green curves, initial $\Delta _t^0$ values are $10^{-4}$ V, $10^{-3}$ V, $10^{-2}$ V, and $10^{-1}$ V, respectively. (Color Online.)

Figure 10 illustrates this asymptotic behavior of $\langle \Delta _{t}\rangle$ as the number of iterations increases for both distributions within the range $q_1 = 2.0$ V and $q_2 = 2.53$ V, in the chaotic regime for four different $\Delta _t^0$ values, such as $10^{-4}$ V, $10^{-3}$ V, $10^{-2}$ V, and $10^{-1}$ V, respectively, within the region where their values alternate between zero and non-zero with time. Remarkably, it is observed that the ensemble averages of $\Delta _t$ for any initial separations reach a steady state non-zero value after a finite number of iterations, which we denote as $\Delta _\textrm{sat} = \langle \Delta _\mathrm{t\rightarrow \infty }\rangle$.

So, the two figures in Fig. 10 demonstrate that even though the separation between the two state variables toggles between zero and non-zero values, the ensemble average of separation for both distributions ultimately saturates to specific values independent of $\Delta _t^0$. The saturation values, $\Delta _\textrm{sat}$ for uniform and symmetric triangular distributions are almost the same, about 0.3 V and 0.4 V, respectively.

**Fig. 11:**
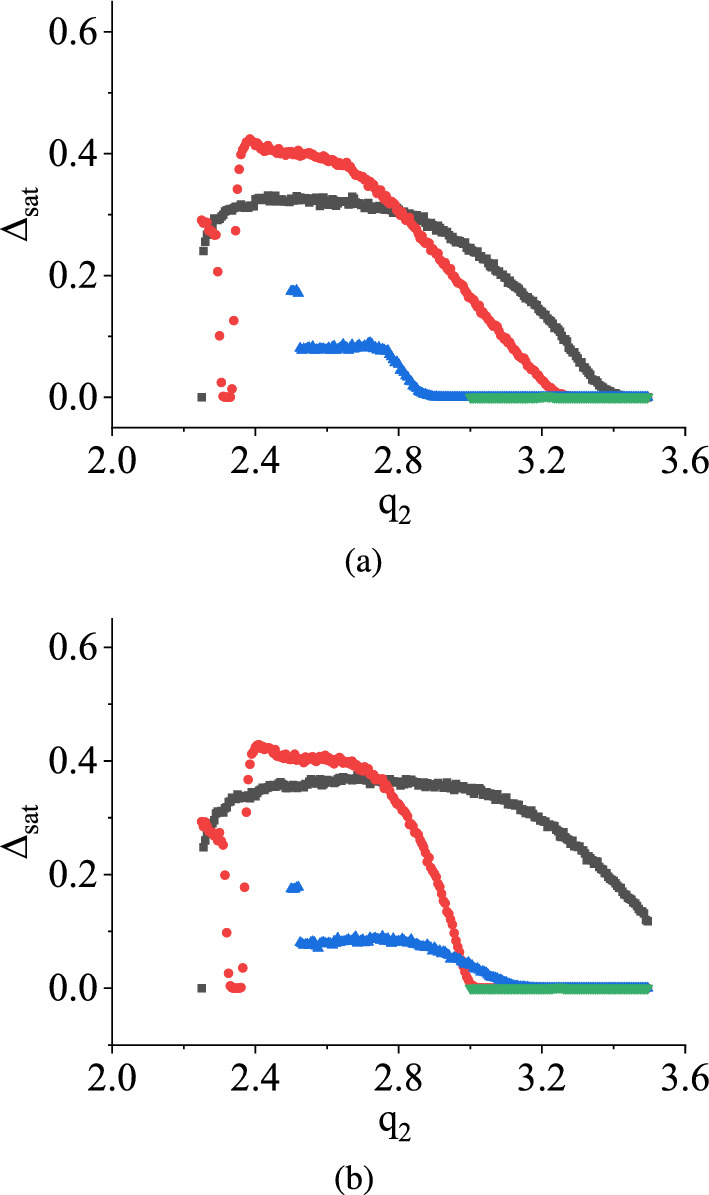
TM method results: The variation of saturation values of damage $\Delta _\textrm{sat}$ in V with $q_2$ in V having $q_1$ is fixed at different values in the case of **a** uniform distribution and **b** symmetric triangular distribution. $q_2$ is varied in between $q_1$ V and 3.5 V. The different color denotes the different fixed values of $q_1$. The black curve denotes when $q_1 = 2.0$ V, the red curve denotes for $q_1 = 2.25$ V, the blue curve denotes for $q_1 = 2.5$ V, and the green curve corresponds to $q_1 = 3.0$ V. (Color online.)

Figure 11a and b depict the saturation values of damage, denoted by $\Delta _\textrm{sat}$, with the variation of $q_2$ for both uniform and symmetric triangular distributions, while maintaining $q_1$ at 2.0 V (black curve), 2.25 V (red curve), 2.5 V (blue curve), and 3.0 V (green curve), respectively. It is evident from both figures that $\Delta _\textrm{sat}$ initially maintains a non-zero value and then decreases with increasing values of $q_2$, while $q_1$ is held constant. Eventually, $\Delta _\textrm{sat}$ reaches zero after surpassing a certain threshold value of $q_2$. This observation suggests that the chaotic behavior of the system transforms into periodic behavior when $\Delta _\textrm{sat}$ reaches zero from a non-zero value after a specific $q_2$ value for uniform and symmetric triangular distributions. However, it is worth noting that the threshold value of $q_2$ for a fixed $q_1$ is distinct for each distribution. An essential finding from both graphs is that as $q_1$ increases, $\Delta _\textrm{sat}$ approaches zero more rapidly, and when $q_1 = 3.0$ V, $\Delta _\textrm{sat}$ is always zero irrespective of $q_2$. This implies that reducing the separation between $q_1$ and $q_2$ by approaching $q_1$ towards $q_2$ leads to a faster transition of the stochastic map into a deterministic state.

**Fig. 12:**
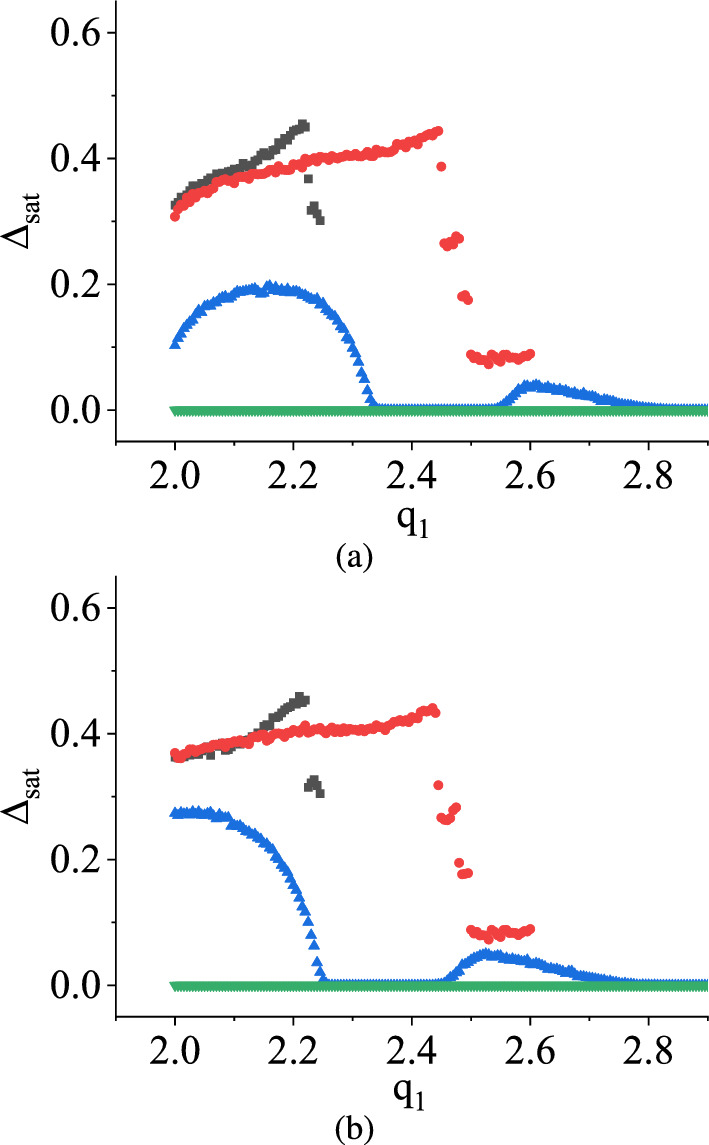
TM method results: The variation of saturation values of damage $\Delta _\textrm{sat}$ in V with $q_1$ in V having $q_2$ is fixed at different values in the case of **a** uniform distribution and **b** symmetric triangular distribution. $q_1$ is varied in between 2.0 V and $q_2$ V. The different color denotes the different fixed values of $q_2$. The black curve denotes when $q_2 = 2.25$ V, the red curve denotes for $q_2 = 2.5$ V, the blue curve denotes for $q_2 = 3.0$ V, and the green curve corresponds to $q_2 = 3.5$ V. (Color online.)

Moreover, in Fig. 12a and b, for both distributions, when $q_2$ is fixed at various values ($q_2$ values are 2.25 V, 2.5 V, 3.0 V, 3.5 V), and $q_1$ is varied within the range of 2.0 V to $q_2$, the parameter $\Delta _\textrm{sat}$ exhibits irregular patterns. In the case where $q_2 = 3.5$ V, $\Delta _\textrm{sat}$ consistently remains at zero for all values of $q_1$, indicating a system-wide periodic behavior across the parameter space. However, in the case of a deterministic map, we get a combination of periodic and chaotic behavior of the state variable in that parameter range. For other $q_2$ values, $\Delta _\textrm{sat}$ switches between zero and non-zero values as $q_1$ gradually approaches the $q_2$ values. This toggling effect lacks periodicity with respect to $q_1$.

Consequently, the stochastic map demonstrates a hopping phenomenon between periodic and chaotic waveforms with variations in $q_1$.

In the next subsection, we have shown analytically why $\Delta _\textrm{sat}$ gives a zero value whenever the two-time series obtained for the TM method merge.

#### Analytical form of $\Delta _{\textrm{sat}}$ for TM method

In the case of the traditional method (TM), the stochastic map *g* is applied for the initial value $V_\textrm{C0}$ and its perturbation value ($V_\textrm{C0} + \Delta _t^0$) with the same random parameter chosen from identical distribution for each iteration. After *n* number of iterations, the values of the state variable and its perturbation will be mapped as $g^n(V_\textrm{C0})$ and $g^n(V_\textrm{C0} + \Delta _t^0)$. The ensemble average of absolute distance between the two values of the state variables will be defined as $\Delta _{\textrm{sat}}$. The ensemble average has to be taken against the time-invariant probability distribution of $p_t(V_C)$. As $\Delta _{\textrm{sat}}$ is the ensemble average of the absolute distance of $g^n(V_\textrm{C0})$ and $g^n(V_\textrm{C0} + \Delta _t^0)$, the functional form $\Delta _{\textrm{sat}}$ in terms of root mean square average [37] can be written as

7 $$\begin{aligned} \Delta _{\textrm{sat}} = \sqrt{\langle (g^n(V_\textrm{C0}+\Delta _t^0)-g^n(V_\textrm{C0}))^{2}\rangle } \end{aligned}$$

The function is analyzed $\langle (g^n(V_\textrm{C0}+\Delta _t^0)$ - $g^n(V_\textrm{C0}))^{2}\rangle$ to check its behavior under different conditions.

8 $$\begin{aligned} \begin{aligned}&\langle (g^n(V_\textrm{C0}+\Delta _t^0)-g^n(V_\textrm{C0}))^{2}\rangle \\&=\quad \langle ((g^n(V_\textrm{C0}+\Delta _t^0)^2+\\&(g^n(V_\textrm{C0})^2-g^n(V_\textrm{C0}+\Delta _t^0)g^n(V_\textrm{C0}))\rangle \\&\quad = \langle (g^n(V_\textrm{C0}+\Delta _t^0)^2\rangle + \langle (g^n(V_\textrm{C0})^2\rangle \\&\qquad \quad \langle -2g^n(V_\textrm{C0}+\Delta _t^0)g^n(V_\textrm{C0})\rangle \end{aligned} \end{aligned}$$

Let us discuss these three terms individually. From the first term:

9 $$\begin{aligned} \langle (g^n(V_\textrm{C0}+\Delta _t^0)^2\rangle =\int (g^n(V_\textrm{C0}+\Delta _t^0)^2 p(V_C) dV_C \end{aligned}$$

We know that as $p(V_C)$ would be same as $p(V_C+\Delta )$ because as $t\rightarrow \infty$, $p_t(V_C)$ and $p_t(V_C+\Delta _t^0)$ would achieve the same stationary form $p(V_C)$.

We can also write the above equation as

10 $$\begin{aligned} \begin{aligned}&\langle (g^n(V_\textrm{C0}+\Delta _t^0)^2\rangle \\&\quad =\int (g^n(V_\textrm{C0}+\Delta _t^0)^2 p(V_C+\Delta _t^0) dV_C\\&\quad = \int (g^n(V_\textrm{C0}+\Delta _t^0)^2 p(V_C+\Delta _t^0) d(V_C+\Delta _t^0)\\&\quad = \int (g^n(z)^2 p(z) d(z)\\ \end{aligned} \end{aligned}$$

Which is the same as the second term.

From the third term, we can write,

11 $$\begin{aligned}&\langle -2g^n(V_\textrm{C0}+\Delta _t^0)g^n(V_\textrm{C0})\rangle \nonumber \\&\quad =-2\langle g^n(V_\textrm{C0}+\Delta _t^0)g^n(V_\textrm{C0})\rangle \end{aligned}$$

There are two different cases for the third term.

1. Case 1: assuming $g^n(V_\textrm{C0}+\Delta _t^0){\mathop {=}\limits ^{n\rightarrow \infty }}g^n(V_\textrm{C0})$ (Non-chaotic regime), then, it can be written as
  12 $$\begin{aligned} \begin{aligned} \langle -2g^n(V_\textrm{C0}+\Delta _t^0)g^n(V_\textrm{C0})\rangle =-2\langle g^n(V_\textrm{C0})^2\rangle \end{aligned} \end{aligned}$$
  If we put this expression in Equation (8), we can see that $\Delta _{\textrm{sat}}$ is zero.
2. Case 2: $g^n(V_\textrm{C0}+\Delta ){\mathop {\ne }\limits ^{n\rightarrow \infty }}g^n(V_\textrm{C0})$ (chaotic regime) and $\Delta _{\textrm{sat}}$ cannot be 0.

### Results for NVN method

As previously stated, for the NVN (Nature versus Nurture) method, the process commences by considering two initially identical values of $V_\textrm{C}$, which then evolve independently. These two evolutions follow distinct sets of random control parameters drawn from identical distributions. Control parameter selection is made from uniform and symmetric triangular distributions.

#### Non-chaotic regime

In the NVN method, due to the utilization of identical initial conditions in conjunction with distinct randomly chosen bifurcation parameters for each iteration, it is observed that the average separation $\langle \Delta _{t}\rangle$ between the state-variable and its perturbation cannot reach zero. This condition prevents the merging of the state variable and its perturbation, which implies the absence of periodic behavior. Consequently, the absence of a non-chaotic regime is observed in the system. The inherent nature of the NVN method, with its specific parameter selection strategy, results in the persistence of chaotic dynamics throughout the simulation.

#### Chaotic regime

**Fig. 13:**
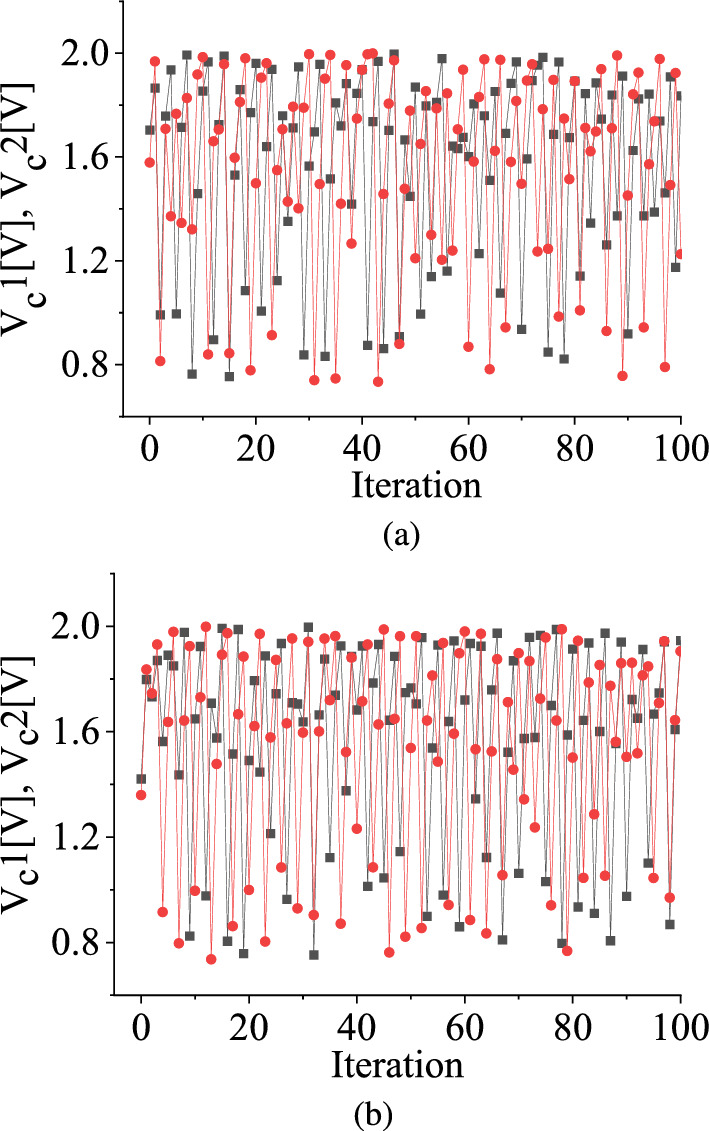
NVN method results: $V_\textrm{C}1$ (black in color) and $V_\textrm{C}2$ (red in color) against the iteration number n **a** for uniform distribution and **b** for symmetric triangular distribution having the range between 2.0 V and 2.53 V. (Color online.)

In Fig. 13, we observe the evolutions of two state variables over iteration using the NVN method for both distributions ranging between 2.0 V to 2.53 V. The two figures clearly demonstrate that the state variables do not merge as iteration progresses. Consequently, the ensemble average $\langle \Delta _{t}\rangle$ of the state variables never reaches zero but tends to approach a constant value with the iteration n. These average values remain consistent for both distributions, similar to the trend observed for TM (Traditional Method).

This behavior further reinforces that the NVN method sustains chaotic dynamics throughout its evolution, regardless of the chosen distribution. The inability of the state variables to merge and the persistent non-zero average separation signifies the continuous presence of chaotic behavior without any periodic regimes in the system.

**Fig. 14:**
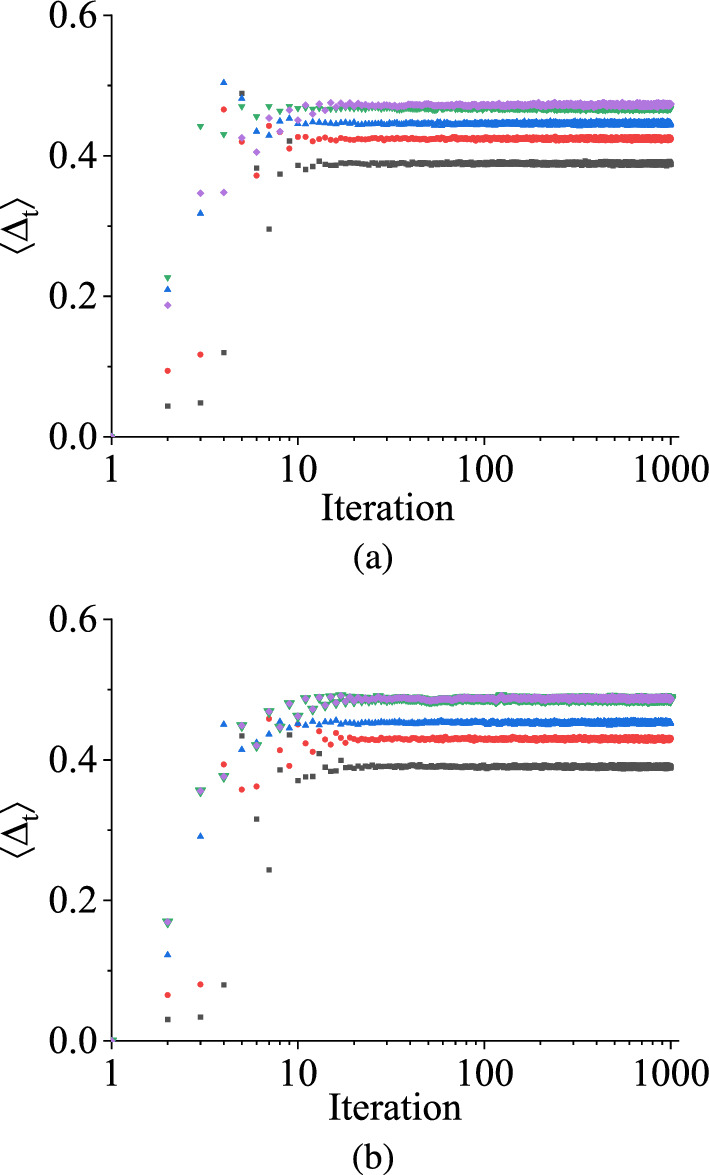
NVN method results: The ensemble average of the damage value, $\Delta _{t}$ in V with the number of iteration n, for various $q_1$ and $q_2$ values in the case of **a** uniform distribution, and **b** symmetric triangular distribution. For each of the figures, the pink color denotes $q_1 = 2.25$ V and $q_2 = 3.5$ V, green color denotes $q_1 = 2.0$ V and $q_2 = 3.5$ V, blue color denotes $q_1 = 2.0$ V, and $q_2 = 3.0$ V, red color denotes $q_1 = 2.0$ V, and $q_2 = 2.53$ V, and black color denotes $q_1 = 2.0$ V and $q_2 = 2.25$ V. (Color online.)

In Fig. 14, the evolution of damage, $\Delta _\textrm{t}$, are presented over the number of iteration for two distributions with five different $q_1$ and $q_2$ values from where the random parameters are chosen using the NVN method. The different combinations of $q_1$ and $q_2$ values are shown in different colors. The plots of $\Delta _\textrm{sat}$ for (2.25 V, 3.5 V), (2.0 V, 3.5 V), (2.0 V, 3.0 V), (2.0 V, 2.53 V), (2.0 V, 2.53 V) combinations are drawn in pink, green, blue, red, and black colors, respectively. The damage values corresponding to various combinations of $q_1$ and $q_2$ initially undergo changes from their initial values and then settle into constant values, i.e., they saturate. These saturation values of $\Delta _\textrm{t}$ remain non-zero and differ between the parameter ranges of the two distributions. However, it is noteworthy that despite the differences in parameter ranges, the saturation values are identical for both distributions in the same parameter ranges, as seen in Fig. 14. Not only that, as the difference between $q_2-q_1$ decreases, $\Delta _\textrm{sat}$ also decreases for both the distribution. The persistent non-zero saturation of $\Delta _\textrm{t}$ reinforces the presence of chaotic behavior in the system, as the damage values never tend to zero but reach stable non-zero values for both distributions. This observation aligns with the absence of a non-chaotic regime in the NVN method, as the chaotic dynamics dominate the behavior of the system irrespective of the parameter distributions.

**Fig. 15:**
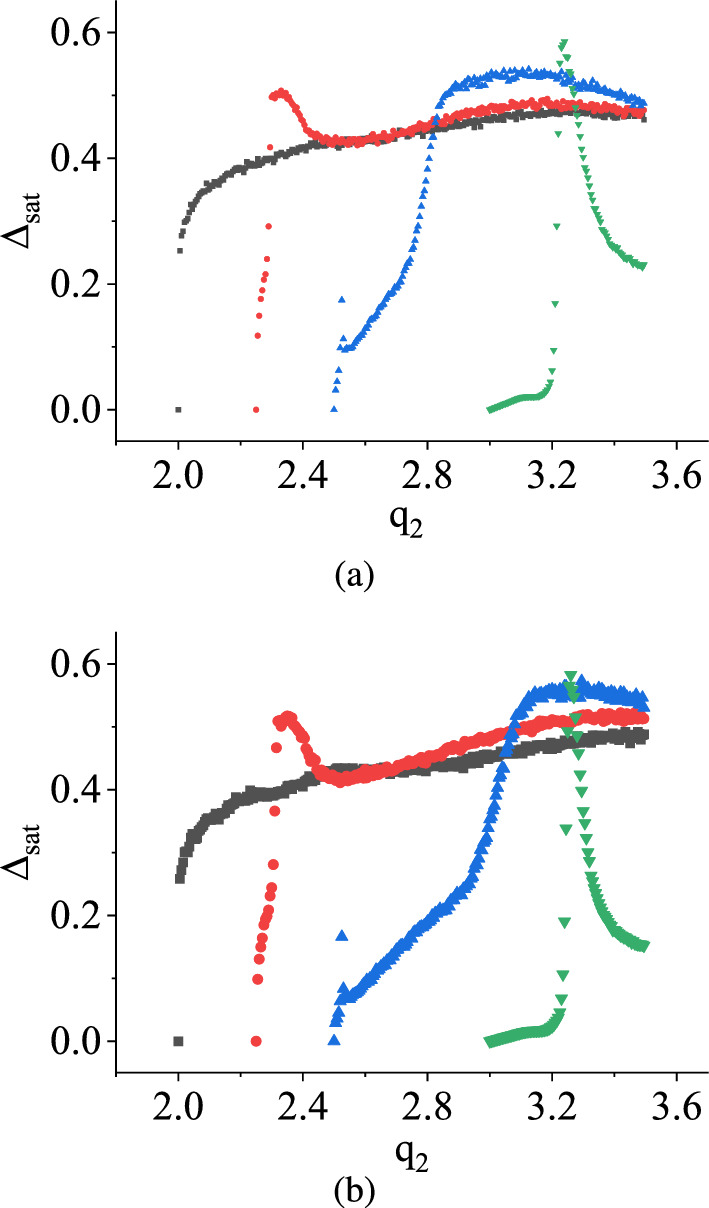
NVN method results: The saturation values of damage, $\Delta _\textrm{sat}$, in V with the variation of $q_2$ in V for different fixed values of $q_1$ in the case of **a** uniform distribution and **b** symmetric triangular distribution. The curves having different colors denote the evolutions of $\Delta _\textrm{sat}$ for different values of $q_1$. $q_2$ is varied in between $q_1$ and 3.5 V. The black curve denotes $q_1 = 2.0$ V, the red curve denotes $q_1 = 2.25$ V, the blue curve denotes $q_1 = 2.5$ V, and the green denotes $q_1 = 3.0$ V. (Color online.)

In Fig. 15, the variations of the saturation value of damage, denoted as $\Delta _\textrm{sat}$, are depicted with respect to the parameter $q_2$, while $q_1$ is kept fixed at different values for both uniform and symmetric triangular distributions. We have chosen $q_1$ values in the Fig. 15 as 2.0 V, 2.25 V, 2.5 V, and 3.0 V, respectively. The curves for different $q_1$ values are plotted in different colors. The figures illustrate that as the value of $q_2$ increases from $q_1$ towards 3.5 V, $\Delta _\textrm{sat}$ increases from zero. This observation indicates that for each fixed value of $q_1$, the stochastic map transitions from a periodic behavior to a chaotic regime.

An interesting behavior is observed when the fixed value of $q_1$ is set to 2.0 V. In this case, $\Delta _\textrm{sat}$ increases from zero and eventually stabilizes at a fixed value close to 0.4 V as $q_2$ is iterated forward. However, as the fixed value of $q_1$ is increased from 2.0 V, the variations of $\Delta _\textrm{sat}$ first reach maximum values from zero as $q_2$ varies and then start to decrease. Each $\Delta _\textrm{sat}$ has a distinct peak value before it begins to decrease in different fashions. This observation implies that as the fixed value of $q_1$ changes, the variations of $\Delta _\textrm{sat}$ exhibit abrupt changes with respect to $q_2$, and there is no apparent correlation between the curves.

In the context of the TM method, as shown in Fig. 11, under the specified condition, $\Delta _\textrm{sat}$ exhibited a state of constancy initially until reaching a certain threshold of $q_2$, after which it progressively diminished to zero with further increments in $q_2$. At $q_1 = 3.0$ V, $\Delta _\textrm{sat}$ is always zero for all the values of $q_2$. Conversely, when applying the NVN method, $\Delta _\textrm{sat}$ displayed entirely unpredictable and irregular patterns of behavior under the same condition. In the context of the TM method, $\Delta _\textrm{sat}$ tends to decrease from a non-zero value to zero. Conversely, in the case of the NVN method, this behavior is reversed.

**Fig. 16:**
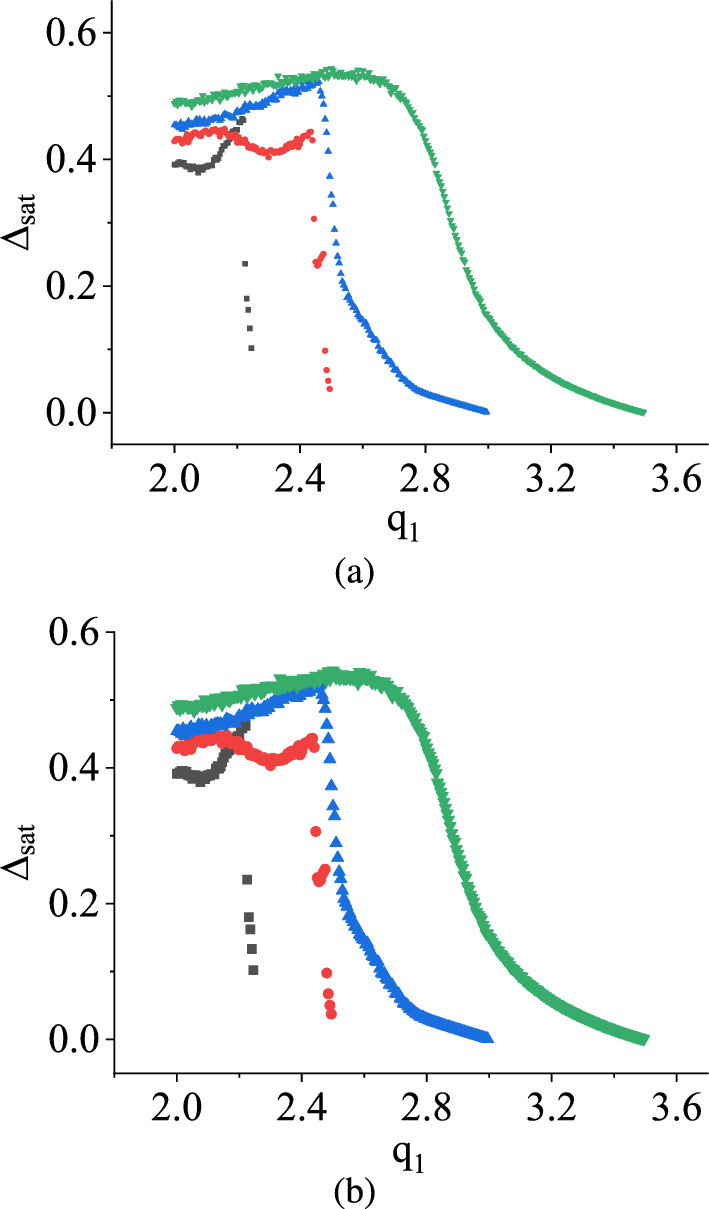
NVN method results: The variations of the saturation values of damage $\Delta _\textrm{sat}$ in V with $q_1$ in V when $q_2$ is fixed at different values for **a** uniform distribution **b** symmetric triangular distribution. Different colors in each figure denote different fixed values of $q_2$. $q_1$ is varied in between 2.0 V and $q_2$. The black color denotes the variation of $\Delta _\textrm{sat}$ for $q_2=2.25$ V, the red color is for $q_2=2.5$ V, the blue color is for $q_2 = 3.0$ V, and the green color is for $q_2 = 3.5$ V. (Color online.)

The variations of the saturation of damage $\Delta _\textrm{sat}$ with $q_1$ by fixing $q_2$ at 2.25 V, 2.5 V, 3.0 V, and 3.5 V for uniform and symmetric triangular distributions are plotted next, as shown in Fig. 16a and b, respectively. Here, for all the curves, $\Delta _\textrm{sat}$ have certain non-zero values up to certain ranges and then attenuate towards zero values while $q_1$ varies in the positive direction. This implies that in this condition, when $q_1$ is varied, and $q_2$ is fixed to a particular value, the stochastic map shows a transition to an ordered behavior from the chaotic ones. With the increase of $q_2$, $q_1$ takes more value to attenuate $\Delta _\textrm{sat}$ towards zero values.

In contrast to the NVN method, when the same conditions are applied to the TM method, as shown in Fig. 12, $\Delta _\textrm{sat}$ remains consistently at zero at $q_2 = 3.5$ V, but erratic behaviors for other $q_2$ values, suggesting a significant difference in behavior between the two methods. In the NVN method, the value of $q_1$ at which the transition occurs from the chaotic dynamics to periodic behavior increases with an increase in the value of $q_2$ in V. Conversely, this transition point decreases under the same conditions in the TM method.

The random, non-smooth one-dimensional map exhibits distinct dynamical behaviors in the saturation value of damage ($\Delta _\textrm{sat}$) when various types of probability distributions are employed. This similar non-conformity of behavior for two dissimilar distributions was observed in the context of a stochastic smooth map, as also demonstrated in the case of a logistic map [17], even though the same methods have been employed to assess the ergodicity and deterministic behavior of the maps.

## Conclusions

This paper investigates the influence of stochasticity on a piecewise-smooth map (2) derived from an inductorless switching circuit, which significantly impacts various applications in chaotic communications. Initially, we have shown that by choosing a specific set of parameter values, the map exhibits a reverse period-incrementing cascade with chaotic inclusions, a bifurcation phenomenon unique to non-smooth systems. By randomly selecting the bifurcation parameter $V_1$ from uniform and triangular distributions, we observe various behaviors of the state variable $V_\textrm{C}$. Depending on the parameter distribution ranges and initial conditions, the system exhibits fully ergodic or toggling between ergodic and non-ergodic dynamics. Notably, despite the presence of chaotic waveforms in $V_\textrm{C}$, the ensemble average of the separation of variables converges to zero over time.

We analyze the probability density function of the state variable of the stochastic piecewise-smooth map within a parameter range randomly chosen from a distribution characterized by $q_1 = 2.0$ V and $q_2 = 3.5$ V. This specific parameter region demonstrates the phenomenon of reverse period-incrementing cascade bifurcation observed in the case of the deterministic map. This curve signifies that the probability of finding the state variable is mostly on the switching manifold. Importantly, the probability density function remains invariant regardless of the chosen initial conditions and the type of distribution within the fixed bifurcation parameter range.

Regarding the deterministic smooth maps, we anticipate chaotic behavior after passing through cascading of periodic attractors in parameter space [42, 47], known as the period-doubling bifurcations. Interestingly, our selected map showcases an interplay between periodic orbits and chaos under specific parameter settings, and the periodicity in each window is greater than the previous by one. We find that the introduction of stochasticity in the switching circuit leads to nonchaotic behavior ($\Delta _t$ tending to zero as iteration progresses) within the parameter space for both distributions. Furthermore, periodic behavior emerges in the parameter space in the stochastic regime, even though the deterministic map exhibits chaos within the same range. In the nonchaotic regime, the separations between the maximum and minimum values of the state variable follow the relationship $\langle \Delta _t\rangle \propto {(\Delta _t^0)^\alpha } \exp (\lambda t)$, which differs from the behavior of the deterministic map. At that instant, we determine the Lyapunov exponent ($\lambda$), the separation of two nearby trajectories, and observe its unconventional dependence on the asymmetry of the distribution.

Additionally, we investigate the TM and NVN methods and examine the transitions from chaos to periodic behavior within the parameter space defined by the ranges of $q_1$ and $q_2$. The traditional method exhibits periodic behavior from chaos around $q_2 \approx 3.2$ V when $q_1$ is fixed at 2.0 V. In contrast, chaos does not occur when $q_2$ is fixed, and $q_1$ is varied, suggesting a different behavior. Notably, the NVN method reveals a transition from chaotic to periodic behavior when $q_2$ is fixed, and $q_1$ varies. Surprisingly, chaos emerges instead of transitioning to periodic behavior when $q_1$ is fixed, and $q_2$ is varied. For different fixed values of $q_1$, the variations of $\Delta _\textrm{sat}$ with $q_2$ do not follow any pattern. It implies that the variation of $\Delta _\textrm{sat}$ entirely depends on the type of method to introduce the stochasticity.

In the TM method, the damage $\Delta _t$ oscillates between zero and nonzero values in a chaotic regime over iteration, indicating varying separations between two state variables. However, attempts to establish correlations with the ratios of two consecutive zero and nonzero separations yield random results, unlike the convergence to a fixed value observed in the deterministic case. This proves that the system shows stochastic behavior due to the randomness in the parameter values.

Our work also confirms findings of previous studies suggesting the independent nature of the TM and NVN methods [37, 48]. This work highlights the determination of saturation values for damage under varying $q_1$ and $q_2$, confirming the consistency of these methods. Notably, we observe a chaotic regime in the NVN method when $q_1$ and $q_2$ differ, reminiscent of the phenomenon of damage spreading in the opinion dynamics model [37].

The chaos generating circuit considered in this study is advantageous for practical implementations, particularly for chaos communication and experimental observations of different border collision bifurcation phenomena in an electronic circuit. For such applications, meticulous determination of parameter values often poses a considerable challenge. There are also ripples in addition to the precise values of parameters. Consequently, the results from this piecewise-smooth map in terms of the expected dynamical responses are useful.

In scenarios involving a deterministic piecewise-smooth map, one may encounter various unconventional bifurcation behaviors that are not observable in smooth maps, known as border collision bifurcations. Certain bifurcations within this context may give rise to potentially hazardous situations in physical systems [49–51] because of its switching phenomena from one functional form to another. Hence, it is crucial to investigate such systems while considering the stochastic nature of their functional map forms and determining the transition points from ergodic to non-ergodic behavior. This work also presents a period-incrementing cascade with chaotic inclusions amidst periodic attractors. This type of bifurcation can not occur in smooth systems. Consequently, comprehending the dynamics of physical switching systems necessitates an exploration of noise effects on these systems.

In future, this work can be extended to investigate the dynamics of a different class of non-smooth map (both piecewise-smooth map and discontinuous piecewise-smooth map) characterized by various border collision bifurcations and subsequently compare the observed dynamics with those obtained from the map employing the idea in our current study.

## Data Availability

All codes implemented in this paper are available upon reasonable request from the corresponding author.
